# Regulatory roles of external cholesterol in human airway epithelial mitochondrial function through STARD3 signalling

**DOI:** 10.1002/ctm2.902

**Published:** 2022-06-09

**Authors:** Liyang Li, Yifei Liu, Xuanqi Liu, Nannan Zheng, Yutong Gu, Yuanlin Song, Xiangdong Wang

**Affiliations:** ^1^ Department of Pulmonary and Critical Care Medicine, Zhongshan Hospital Fudan University Shanghai Medical College Shanghai China; ^2^ Center of Molecular Diagnosis and Therapy The Second Hospital of Fujian Medical University Quanzhou Fujian China; ^3^ Shanghai Institute of Clinical Bioinformatics Shanghai China; ^4^ Shanghai Engineering Research for AI Technology for Cardiopulmonary Diseases Shanghai China

**Keywords:** airway epithelia, cholesterol, COPD, MFN2, mitochondria, STARD3

## Abstract

**Background:**

Hypercholesterolemia is found in patients with chronic lung inflammation, during which airway epithelial cells play important roles in maintenance of inflammatory responses to pathogens. The present study aims at molecular mechanisms by which cholesterol changes airway epithelial sensitivity in response to smoking.

**Methods:**

Human bronchial epithelial cells (HBEs) were stimulated with cigarette smoke extract (CSE) and mice were exposed to CS/lipopolysaccharide (LPS) as models in vitro and in vivo. Severe COPD patients and healthy volunteers were also enrolled and the level of cholesterol in plasma was detected by metabolomics. Filipin III and elisa kits were used to stain free cholesterol. Mitochondrial function was detected by mitotracker green, mitotracker green, and Seahorse. Mitochondrial morphology was detected by high content screening and electron microscopy. The mRNA and protein levels of mitochondrial dynamics‐related proteins were detected by RT‐qPCR and Western blot,respectively. BODIPY 493/503 was used to stain lipid droplets. Lipidomics was used to detect intracellular lipid components. The mRNA level of interleukin (IL)‐6 and IL‐8 were detected by RT‐qPCR.

**Results:**

We found that the cholesterol overload was associated with chronic obstructive pulmonary disease (COPD) and airway epithelia‐driven inflammation, evidenced by hypercholesterolemia in patients with COPD and preclinical models, alteration of lipid metabolism‐associated genes in CSE‐induced airway epithelia and production of ILs. External cholesterol altered airway epithelial sensitivity of inflammation in response to CSE, through the regulation of STARD3‐MFN2 pathway, cholesterol re‐distribution, altered transport and accumulation of cholesterol, activities of lipid transport regulators and disorder of mitochondrial function and dynamics. MFN2 down‐regulation increased airway epithelial sensitivity and production of ILs after smoking, at least partially by injuring fatty acid oxidation and activating mTOR phosphorylation.

**Conclusions:**

Our data provide new insights for understanding molecular mechanisms of cholesterol‐altered airway epithelial inflammation and for developing diagnostic biomarkers and therapeutic targets to improve patient outcomes.

## Graphical abstract

1. STARD3, upregulated in COPD, could help cholesterol transfer to mitochondria and lead to mitochondrial dysfunction.
2. Exogenous cholesterol reversed the protective MFN2 upregulation in CSE stimulation and exacerbated the mitochondrial dysfunction.
3. Mitochondrial dysfunction caused by MFN2 downregulation altered the lipid homeostasis related to the activation of mTOR.

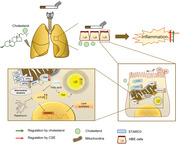

**Abbreviations**
COPD: chronic obstructive pulmonary diseases
MFN: mitofusin
STARD: steroidogenic acute regulatory‐related lipid transfer domain
HBE: human bronchial epithelial cells
OPA1: optic atrophy 1
DRP1: dynamin‐related protein 1
MFF: mitochondrial fission factor
FIS1: mitochondrial fission protein 1
HCD: high cholesterol diet
ND: normal diet
CSE: Cigarette smoke extract
OCR: oxygen consumption rate
SREBP2: sterol‐regulatory element binding protein 2
HMGCR: HMG‐CoA reductase
LDLR: low‐density lipoprotein receptor
DEG: differentially expressed genes
IL: interleukin
MMP: mitochondrial membrane potential
mTOR: mammalian target of rapamycin
LD: lipid droplet
FAO: fatty acid oxidation
ETO: etomoxir
L‐CAR: L‐cartinine
TAG: triacylglyceride

## 1 INTRODUCTION

The airway inflammation is one of the characteristics of chronic obstructive pulmonary disease (COPD), together with airflow obstruction and lung damage, responsible for the progression of the disease.^1^ The exposures to smoking, pollution and pathogens are major causes to stimulate airway epithelial cells and initiate chronic processes of inflammation and injury.^1^, ^2^, ^3^, ^4^, ^5^ Cholesterol was involved in lung inflammation and injury in patients.^2^ Clinical studies demonstrated that levels of cholesterol elevated in the peripheral blood of patients with COPD or smokers and were positively correlated to disease severity.^3^, ^4^, ^5^ The metabolism of lipid molecules altered in patients with COPD, including phospholipids, sphingolipids and fatty acid metabolism,^6^ although the exact mechanism and role of intracellular cholesterol in COPD remained unclear. Preclinical studies on lung 30 cell types, activities and proteomic profiles illustrated that cholesterol biosynthesis increased in lung epithelia and lipofibroblasts and the frequency of airway epithelial cells altered during aging.^7^ The cholesterol‐related metabolisms in lung epithelial cells were considered as cell type‐specific phenomes and potential markers for lung aging. Growing evidence supports a prominent role of lipid metabolism in the development of COPD.^8^ The imbalance of pulmonary lipid homeostasis could drive cigarette smoke (CS)‐induced inflammation, due to the lipid accumulation in pulmonary macrophages.^9^ Of multiple signal pathways and metabolisms, transcriptional changes in cholesterol biosynthesis were dependent upon loss of top‐ranked genes, for example, angiotensin‐converting enzyme 2 receptor.^10^ The exact mechanism and role of cholesterol overload in human bronchial epithelial cells (HBEs) as the first defense against pathogens remain unclear.

Steroidogenic acute regulatory‐related lipid transfer domain protein (STARD) family has 15 members, responsible for intracellular lipid transport. Of those, STARD1, STARD3, STARD4, STARD5 and STARD6 bind cholesterol, facilitate cholesterol transport and maintain cellular cholesterol homeostasis.^11^ STARD3 was reported to be localized on the membrane of late endosomes, regulate cholesterol transport from endosomes to mitochondria and increase secondary accumulation of cholesterol in the outer mitochondrial membrane.^17^, ^18^ STARD3 overexpression increased cholesterol redistribution to the mitochondria, causing over‐production of reactive oxygen species and enhancement of steroidogenesis.^12^, ^13^

Increasing evidence suggests an important role of mitochondrial dysfunction in the pathogenesis and development of lung diseases.^14^ Oxidative stress, mitophagy and mitochondrial dynamics including fusion and fission were found to play crucial roles in COPD and serve as therapeutic targets.^15^, ^16^ Of various metabolic pathways, mitochondria play a key role in lipid metabolism, cholesterol homeostasis and interactions among intracellular organelles,^17^, ^18^, ^19^ although the exact mechanism in COPD remains unclear. The aim of the present study was to investigate alterations of cholesterol metabolism in COPD and potential mechanisms by which cholesterol regulated mitochondrial function in lung epithelial cells. We found that cholesterol overload increased inflammatory response in smoke‐challenged HBEs and initiated mitochondrial dysfunction regulated by STARD3. Down‐regulation of STARD3 could alleviate smoking‐induced inflammation in lung epithelial cells and partly recover the mitochondrial function. Our data indicate that the cholesterol‐related mitochondrial dysfunction and interaction between mitochondrial dysfunction and lipid homeostasis alter epithelial cell suspensibility in inflammatory response and can be an alternative of new therapeutic targets for the disease.

## 2 RESULTS

### 2.1 Efflux of cholesterol into lung epithelial cells enhances cytokines production

**FIGURE 1:**
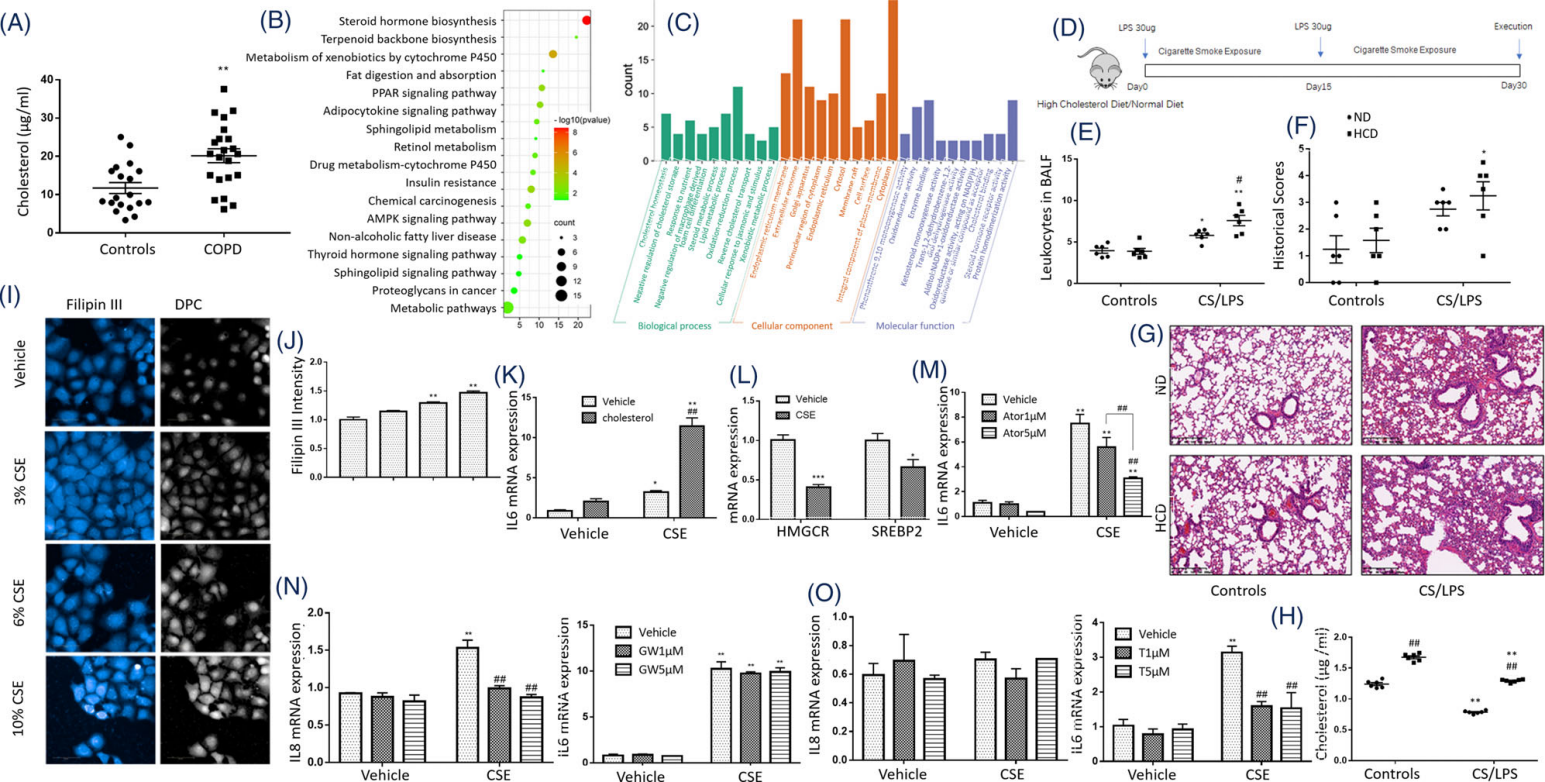
Cholesterol overload and inflammation in cells and animals with smoking and in patients with chronic obstructive pulmonary diseases (COPDs). The cholesterol level was detected by Gas Chromatography‐Mass Spectrometry (GC‐MS)/MS metabolomics in the plasma of COPD patients (*n* = 22) and healthy volunteers (*n* = 19) (A). Kyoto Encyclopedia of Genes and Genomes (KEGG) enrichment (B) and gene ontology (GO) enrichment analysis (C) of differentially expressed genes (DEGs) were calculated among 384 lipid metabolism‐related genes in 6% cigarette smoke extract (CSE)‐stimulated human bronchial epithelial cells (HBEs). The mice were intratracheally administered with lipopolysaccharide (LPS) at 30 mg/0.2 ml/mouse or vehicle on days 0 and 15 and exposed to cigarette smoke (CS) for another 28 days and sacrificed on day 30 (D), including mice treated with vehicle and fed with normal diet (ND) or high cholesterol diet (HCD) as controls, or mice treated with CS/LPS and fed with normal diet or HCD as CS/LPS (four groups/study and six mice/group). The number of leukocytes (E) in the bronchoalveolar lavage fluid (BALF) and histological scores (F) were counted. Histology presents the infiltration of leukocytes into and around the airway, hyperplasia and tissue injury (G). The cholesterol level in BALF was detected by GC‐MS/MS metabolomics (H). Intracellular cholesterol staining with Filipin III and digital phase contrast (DPC) were performed in HBEs treated with 3, 6 and10% CSE for 24 h (I). Mean fluorescence intensity of Filipin III staining was calculated by Perkin‐Elmer Harmony image analysis software (J). mRNA levels of interleukin (IL) 6 were measured in HBEs 24 h after stimulated with 6% CSE and treated with 100 μM cholesterol (K) or atorvastatin (Ator) at 1 or 5 μM (L) 2 h before CSE stimulation. mRNA levels of sterol‐regulatory element binding protein 2 (SREBP2) and 3‐hydroxy‐3‐methyl glutaryl (HMG)‐CoA reductase (HMGCR) were measured in HBEs treated with 6% CSE (L). mRNA levels of IL6 and IL8 were measured in HBEs 24 h after stimulated with 6% CSE and treated with 100 μM cholesterol at GW3965 (GW) at 1 or 5 μM (N) or T0901317 (T) at 1 or 5 μM (O) 2 h before CSE stimulation. *p < .05; **p < .01. * represents the intragroup comparison, and # represents the intergroup comparison

Plasma levels of cholesterol in patients with severe COPD were significantly higher than those in the heathy (Figure 1A, Table S1). To determine the mainly changed lipid metabolism pathways, we screened mRNA levels of the lipid metabolism‐associated gene panel with 384 genes (details are listed in Table S2) in HBEs treated with 6% CS extract (CSE) or vehicle and found 67 differentially expressed genes (DEGs) (Figure S1A,B). Of those DEGs with statistical significance (Figure S1C), steroid hormone biosynthesis and cholesterol‐related pathways were obviously changed (Figure 1B,C). To furthermore evaluate the effect of cholesterol overload in blood on the severity of lung injury and inflammation, mice were fed with high cholesterol diet (HCD) and developed more severe lung inflammation in CS/LPS‐treated mouse models (Figure 1D) than those fed with normal diet (ND). CS/LPS‐treated mice with HCD had more severe lung tissue destruction, inflammatory cell infiltration and elevated total cells in bronchoalveolar lavage fluid (BALF), rather than ND‐treated mice (Figure 1E–G). The cholesterol level in BALF was higher in mice with HCD while decreased after CS/LPS treatment (Figure 1H).

Airway epithelial cells act as the primary receptor in exposure to external pathogens and stimuli and as the secondary initiator to induce local and systemic inflammation by releasing inflammatory mediators. The concentration of CSE at 5%–20% could significantly inhibit the proliferation of HBEs, and the concentration >20% showed significant cytotoxicity, which was validated and shown in Figure S2. We stimulated HBEs with CSE at different concentrations and found that intracellular levels of cholesterol elevated (Figure 1I,J). CSE and cholesterol combination (CSE/cholesterol) induced more severe inflammation in HBEs than CSE alone (Figure 1K). Two key regulators in cholesterol biosynthesis, 3‐hydroxy‐3‐methylglutaryl‐coenzyme A reductase (HMGCR) and sterol‐regulatory element binding protein 2 (SREBP2) were found significantly down‐regulated (Figure 1L). CSE induced higher mRNA level of interleukin (IL)‐6 and IL‐8 in HBEs, which was parallel with published ELISA data as inflammatory characteristics of CSE‐stimulated HBEs^20^ and partially inhibited by administration of atorvastatin in a dose‐dependent pattern (Figure 1M). To evaluate roles of the liver X receptors (LXRs), which induce cholesterol efflux, LXR agonists T0901317 and GW3965 were applied. GW3965 significantly inhibited CSE‐induced up‐expression of IL‐8 (Figure 1N), while T0901317 had more inhibitory effects on CSE‐induced up‐expression of IL‐6 (Figure 1O).

### 2.2 Intracellular cholesterol overload changes mitochondrial transcriptome and oxidative phosphorylation

**FIGURE 2:**
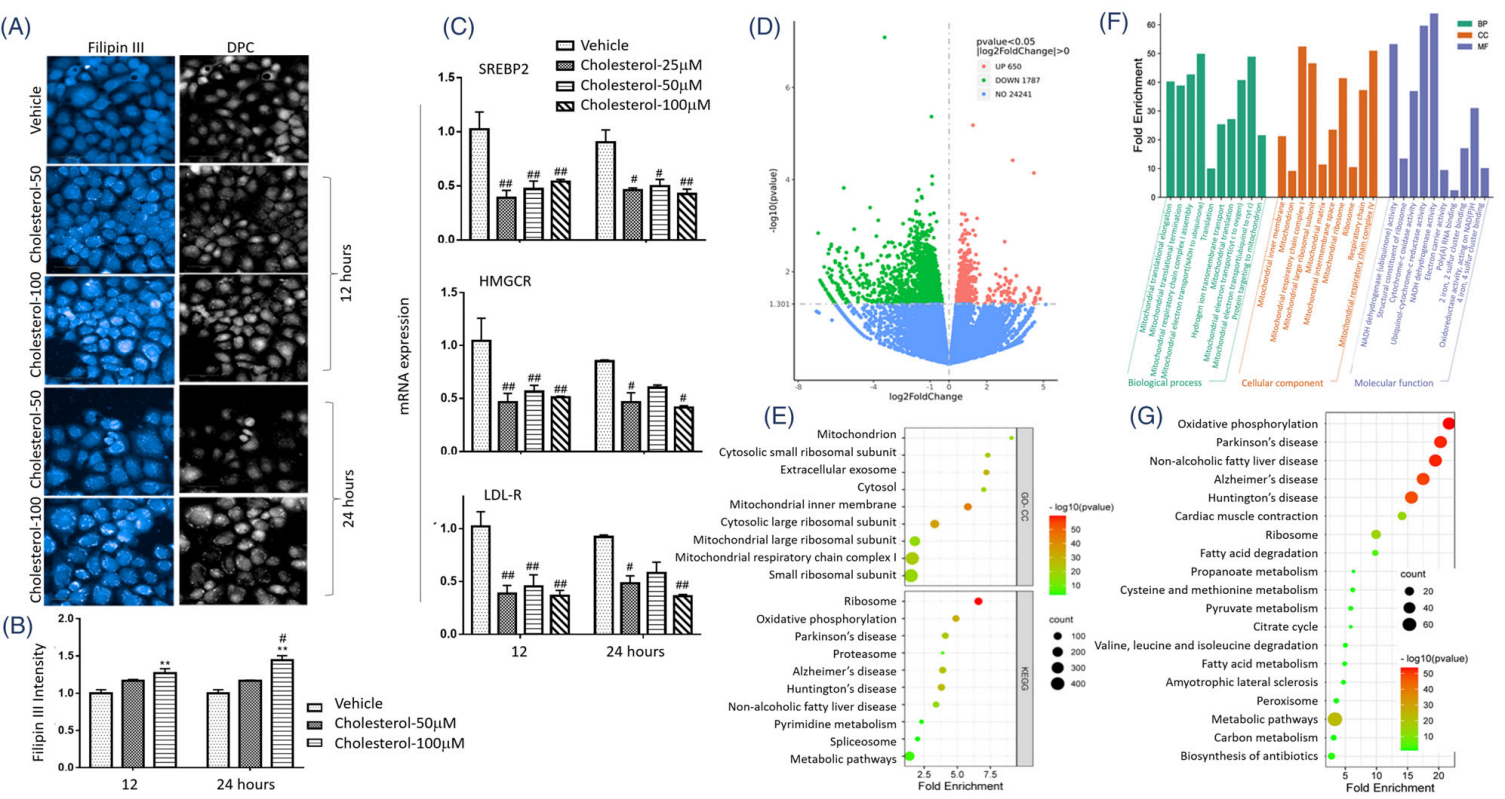
Effects of exogenous cholesterol on human bronchial epithelial lipid accumulation and transcriptomic profiles. Intracellular cholesterol staining with Filipin III and digital phase contrast (DPC) (A) and mean fluorescence intensity (B) were measured in human bronchial epithelial cells (HBEs) treated with cholesterol at 50 or 100 μM for 12 and 24 h. mRNA levels of sterol‐regulatory element binding protein 2 (SREBP2), 3‐hydroxy‐3‐methylglutaryl‐coenzyme A reductase (HMGCR) and low‐density lipoprotein receptor (LDL‐R) were assessed in HBEs stimulated with cholesterol at 25, 50 and 100 μM for 12 or 24 h (C). The volcano map (D) and KEGG and gene ontology (GO) enrichment analysis (E) of RNA‐Seq data was performed in 6% cigarette smoke extract (CSE)‐stimulated HBEs with or without cholesterol. Of those, GO (F) and KEGG (G) enrichment analysis of 242 differentially expressed mitochondrial genes were filtered by MitoCarta3.0 database. *p < .05; **p < .01. * represents the intragroup comparison, and # represents the intergroup comparison

The exogenous cholesterol dynamically increased the accumulation of intracellular cholesterol in HBEs in a dose‐dependent pattern (Figure 2A,B). mRNA expression of cholesterol metabolism genes was down‐regulated 12 and 24 h after adding of exogenous cholesterol, including SREBP2, HMGCR and low‐density lipoprotein receptor (LDLR, Figure 2C). Of transcriptomic profiles, 650 genes were up‐regulated, and 1787 genes were down‐regulated in HBEs with CSE/cholesterol, as compared to those without cholesterol (*p* < .05, |log2Fold Change|>0, Figure 2D). Oxidative phosphorylation (OXPHOS) and metabolic pathways ranked top 10 (Figure 2E). About 4292 DEGs significantly differed between cells with vehicle and cholesterol, 2437 between cells with CSE and CSE/cholesterol and 7214 between cells with cholesterol and CSE/cholesterol, respectively (Figure S3A,B). Figure S3C presented significant differences of mRNA expression between CSE‐stimulated HBEs with vehicle or cholesterol. Of those DEGs, 242 mitochondria‐associated genes had high confidence of mitochondrial localization in CSE‐stimulated HBEs, of which top 50 mitochondria‐associated transcriptional changes were closely related to protein‐protein interactions (Figure S3D,E). Those mitochondria‐associated genes were filtered by MitoCarta3.0 database^21^ and are listed in Table S3. Genes related to mitochondrial inner membrane and respiratory chain significantly changed in cellular component, genes related to mitochondrial translational process and electron transport were in biological process, and genes related to NADH activity in molecular function (Figure 2F). Of those biological processes, the OXPHOS ranked first, and mitochondria‐associated DEGs were also enriched to fatty acid metabolism, citrate cycle and other metabolic pathways (Figure 2G).

### 2.3 Cholesterol enhances mitochondrial damage in exposure to CS

**FIGURE 3:**
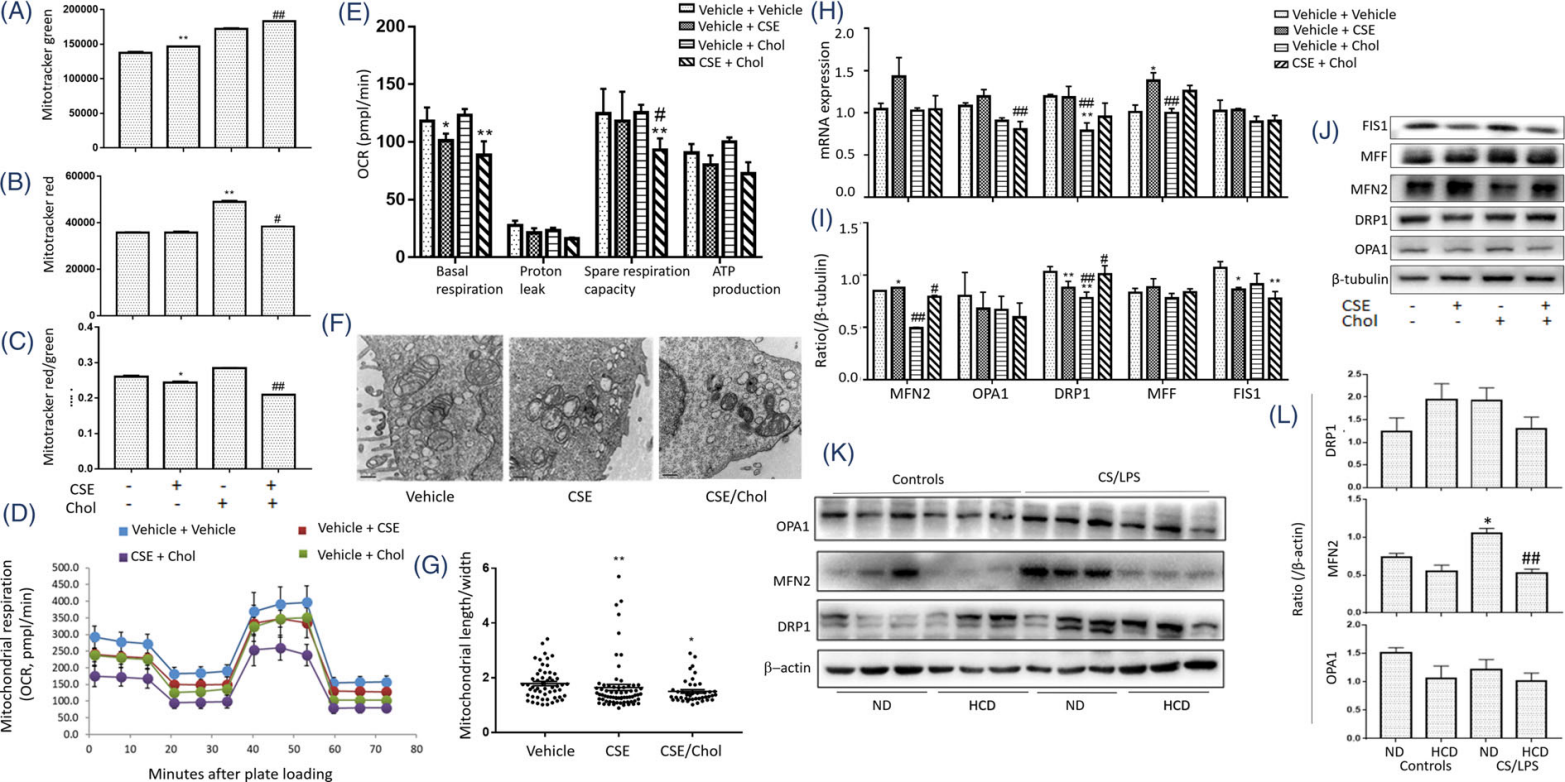
Roles of cholesterol overload in mitochondrial dysfunction. Mitochondrial mass by mitotracker green staining (A), mitochondrial membrane potential mitotracker red staining (B) and standardized mitochondrial membrane potential by mitotracker red/green (C) were measured in human bronchial epithelial cells (HBEs) treated with 6% cigarette smoke extract (CSE), 100 μM cholesterol (Chol) or CSE/Chol combination by flow cytometry. Oxygen consumption rate (OCR) was measured by Seahorse (D) every 10 min for 80 min, representing mitochondrial respiration. Basal respiration, proton leak, spare respiration capacity and ATP production were calculated (E). The changes of mitochondrial morphology were observed by electron microscope (F). The ratio of length/width in each mitochondrial was calculated (G). mRNA (H) and protein levels (I and J) of mitofusin 2 (MFN2), optic atrophy 1 (OPA1), dynamin‐related protein 1 (DRP1), mitochondrial fission factor (MFF) and mitochondrial fission protein 1 (FIS1) were assayed. Protein levels of OPA1, MFN2 and DRP1 in mice were measured (K and L) after treated with vehicle and fed with normal diet (ND) or high cholesterol diet (HCD), or with CS/LPS and fed with ND or HCD (*n* = 6/group). *p < .05; **p < .01. * represents the comparison with vehicle, and # represents the comparison with CSE

To investigate roles of cholesterol in mitochondrial function, we measured the mitochondrial mass and membrane potential (MMP) in HBEs and found that CSE elevated the mitochondrial mass (Figure 3A) and decreased the MMP (Figure 3B,C), as compared with cells with vehicle. Addition of cholesterol increased levels of mitochondrial mass. That increase did not culminate in compensatory improvement of MMP. We evaluated the oxygen consumption rate (OCR) and extracellular acidification rate (ECAR) and found that basal respiration, proton leak, ATP production and spare respiratory capacity significantly decreased in HBEs with CSE, which were exacerbated by cholesterol (Figure 3D,E), while the ECARs were not changed (Figure S4). CSE led to swollen and fragmented mitochondria evidenced by ultrastructural alterations under electron microscope (Figure 3F,G, Figure S5A–C), and the ratio of length/width decreased in HBEs with CSE. Cholesterol exacerbated mitochondrial vacuolization and destroyed mitochondrial cristae and membrane structure (Figure 3F,G).

We analyzed mRNA expression of mitochondrial fusion genes including mitofusin (MFN) 1, MFN2, optic atrophy 1 (OPA1) and mitochondrial fission genes including dynamin‐related protein 1 (DRP1), mitochondrial fission factor (MFF) and mitochondrial fission protein 1 (FIS1) by cell RNA sequencing (RNA‐seq) and verified by RT‐qPCR. Data from HBE RNA‐seq demonstrated that CSE induced low‐expression of MFN2 mRNA and high‐expression of MFF and FIS1, which were altered by adding cholesterol (Figure S5D). In validation, levels of MFN2, OPA1 and MFF mRNA expression were higher in HBEs with CSE than those with vehicle, while cholesterol alone reduced the mRNA expression of OPA1 and DRP1, as compared with vehicle (Figure 3H). Of mitochondrial dynamics‐associated proteins, MFN2 levels in cholesterol‐treated cells were significantly lower than other groups, DRP1 in CSE or cholesterol‐treated cells lower than those with vehicle or the combination, and FIS1 in cells with CSE, cholesterol, or the combination lower than those with vehicle, respectively (Figure 3I,J). Of mitochondrial fission/fusion proteins in the inner and outer membrane of mitochondria (DRP1, MFN2 and OPA1), protein levels of MFN2 in lung tissues harvested from animals were down‐regulated by HCD and up‐regulated by CS/LPS exposure (Figure 3K,L), which were consistent with results from the in vitro studies.

### 2.4 STARD3 regulates epithelial inflammation and mitochondrial function

**FIGURE 4:**
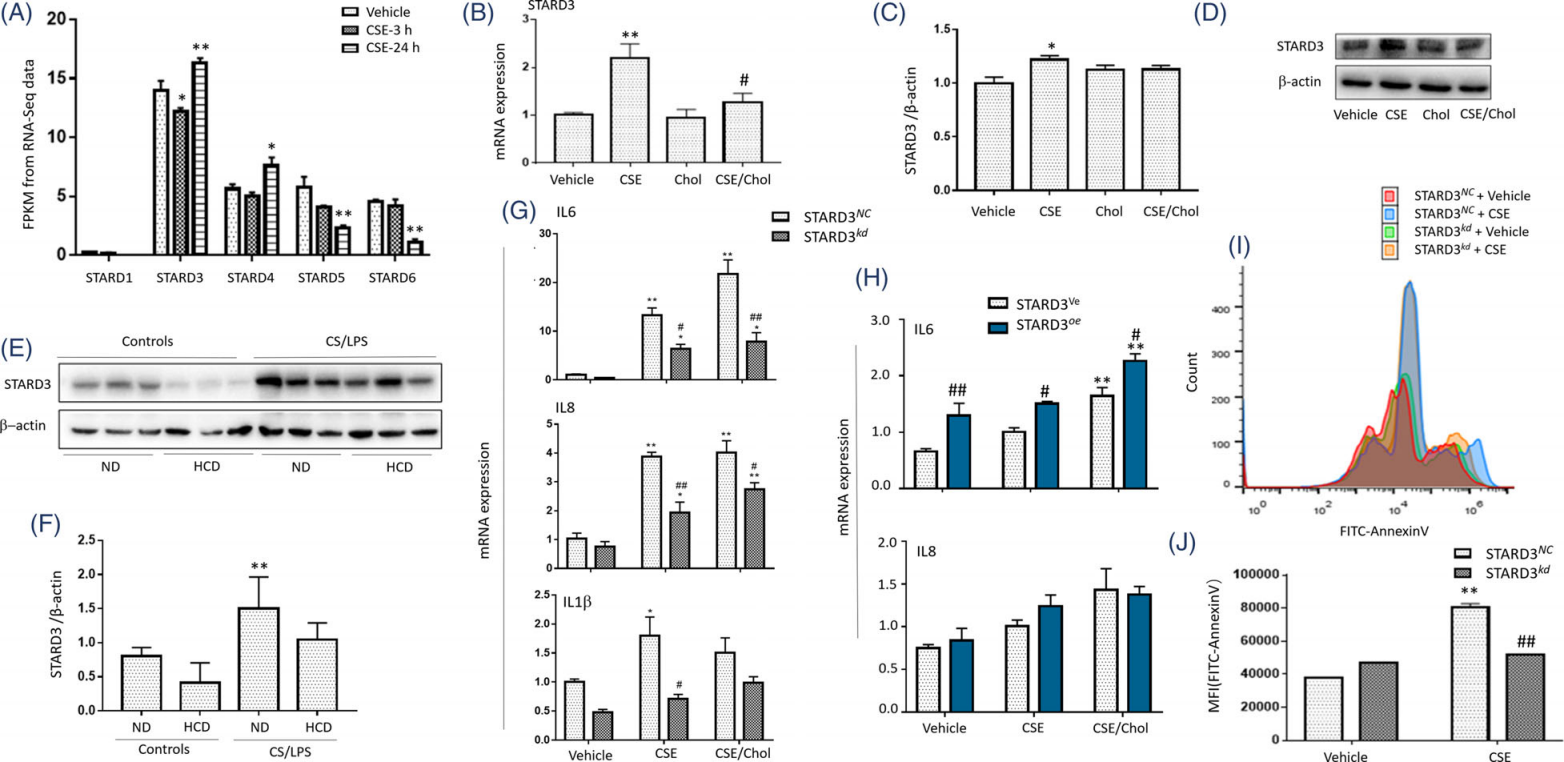
Down‐ or up‐regulation of steroidogenic acute regulatory‐related lipid transfer domain‐3 (STARD3) in epithelial inflammation and death. mRNA levels of STARD family members (STARD1, STARD3, STARD4, STARD5 and STARD6) were measured in human bronchial epithelial cells (HBEs) by RNA‐seq 3 and 24 h after stimulation with 6% cigarette smoke extract (CSE) or vehicle (A). Levels of STARD3 mRNA (B) and protein (C and D) were assessed in HBEs stimulated with vehicle, 6% CSE, 100 μM cholesterol (Chol) and CSE/Chol combination. Protein levels of STARD3 in mice (E and F) were measured after treated with vehicle and fed with normal diet (ND) or high cholesterol diet (HCD), or with CS/LPS and fed with ND or HCD (*n* = 6/group). mRNA levels of IL6, IL8 and IL1β altered in HBEs with knockdown of STARD3 (STARD3*^kd^*) or negative control (STARD3*^NC^*) after stimulation with CSE or CSE/Chol (G), while mRNA levels of IL6 and IL8 were measured in HBEs with the plasmid to overexpress STARD3 (STARD3*^oe^*) or control (STARD3*^Ve^*) (H). The counts of cells with Annexin V‐FITC positive staining (I) and the total density of Annexin V‐FITC (L) differed between STARD3*^kd^* or STARD3*^NC^* HBEs treated with 6% CSE. *p < .05; **p < .01. * represents the intragroup comparison, and # represents the intergroup comparison

To explore effects of intracellular cholesterol accumulation on mitochondrial function and inflammation, we measured transcriptomic profiles of HBEs 3 and 24 h after CSE stimulation. Our RNA‐seq data described that STARD3 was the highest expressed gene among members of STARD family related to cholesterol homeostasis in HBEs (Figure 4A). mRNA levels of STARD3 or STARD4 were significantly lower or higher 3 or 24 h after CSE exposure, respectively (Figure 4A). mRNA expression of STARD5 and STARD6 decreased by time after CSE stimulation, especially at 24 h. Those biological functions of STARD members varied in response to alterations of cholesterol homeostasis in CSE‐induced pathophysiology. We further evaluated transcriptional and translational levels of STARD3 in cells and animals and found that CSE up‐regulated expression of STARD3 mRNA (Figure 4B) and protein (Figure 4C,D), while levels of STARD3 in CSE/cholesterol were significantly lower than those in CSE alone. Protein expression of STARD3 reduced in lung tissues of mice with HCD, as compared with those with ND or with CS/LPS (Figure 4E,F).

In order to define regulatory roles of STARD3 in inflammatory response, we knocked down STARD3 in HBEs with siRNA against STARD3 (STARD3*^kd^*) or over‐expressed STARD3 in HBEs transferred with STARD3 (STARD3*^oe^*) and measured mRNA expression of pro‐inflammatory cytokines. mRNA expression of IL‐6, IL‐8 and IL‐1β (Figure 4G) significantly decreased in STARD3*^kd^*, as compared with negative control cells (STARD3*^NC^*) (*p* < .05 or less, respectively). Levels of IL‐6, IL‐8 and IL‐1β mRNA expression in STARD3*^kd^* with CSE/cholesterol were higher than those with vehicle or CSE alone. Levels of IL‐6 mRNA were significantly higher in STARD3*^oe^*, as compared with negative control cells (STARD3*^Ve^*) (Figure 4H). CSE/cholesterol significantly increased IL‐6 mRNA expression in STARD3*^oe^*, as compared with STARD3*^Ve^* or in STARD3*^oe^* with vehicle or CSE, respectively (Figure 4H). Similar effects of CSE/cholesterol to HBEs were observed in BEAS‐2B cells (Figure S5E,F). We also evaluated roles of STARD3 in apoptosis and found that CSE increased the apoptosis, while STARD3*^kd^* had lower amount of apoptosis after CSE stimulation, as compared with STARD3*^NC^* (Figure 4I,J).

### 2.5 STARD3 down‐regulation plays preventive roles in mitochondrial cholesterol overload

**FIGURE 5:**
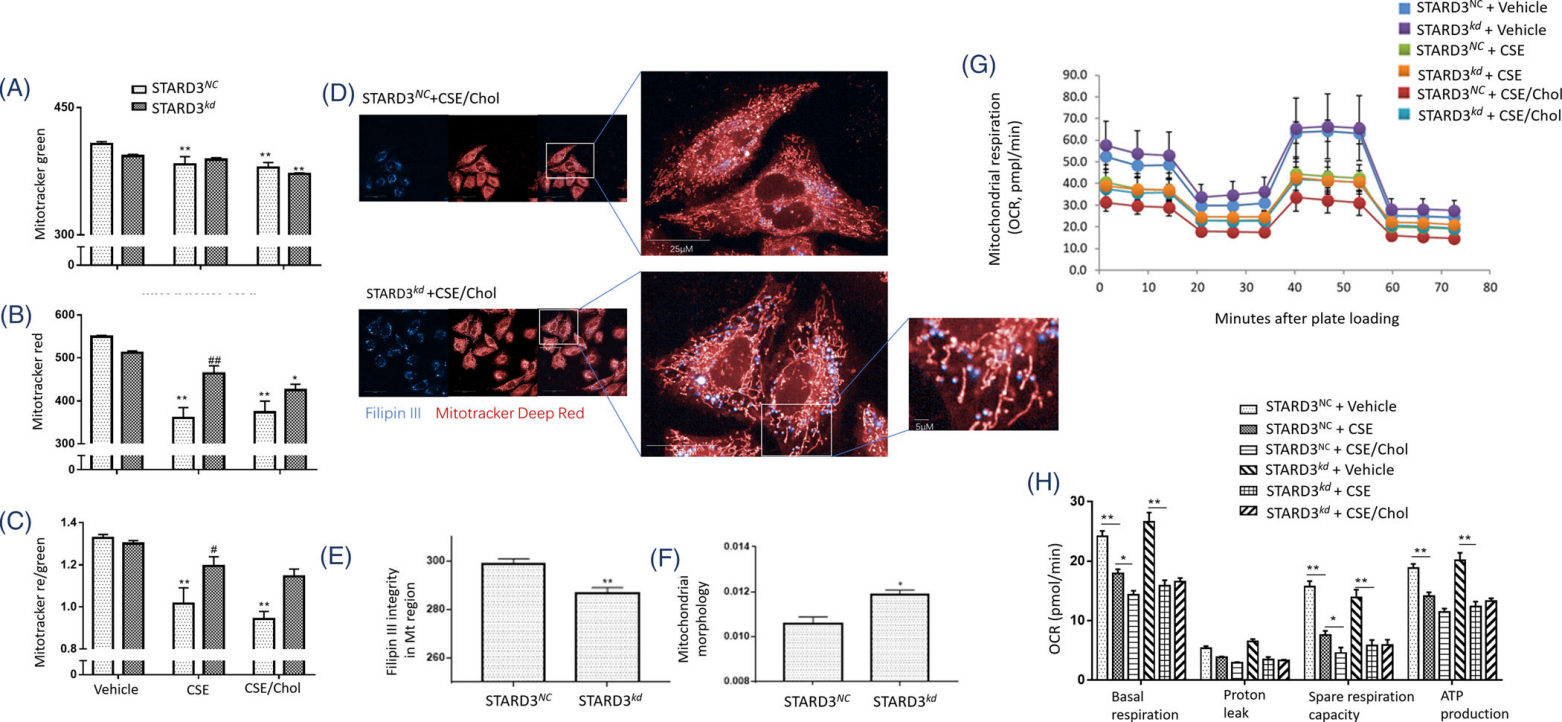
Roles of steroidogenic acute regulatory‐related lipid transfer domain‐3 (STARD3) could affect the mitochondrial function and cholesterol transport. Mitochondrial mass by mitotracker green staining (A), mitochondrial membrane potential mitotracker red staining (B) and standardized mitochondrial membrane potential by mitotracker red/green (C) were measured in STARD3*^kd^* or STARD3*^NC^* treated with 6% cigarette smoke extract (CSE), 100 μM cholesterol (Chol) or CSE/Chol combination. The cholesterol and mitochondria within STARD3*^kd^* or STARD3*^NC^* were stained with Filipin III and localized by mitotracker deep red and then scanned by high content screening with 63X water object and confocal mode (D). The intensity of Filipin III (E) and scores of mitochondrial morphologies (F) were calculated in STARD3*^kd^* or STARD3*^NC^* with Perkin‐Elmer Harmony image analysis software. Mitochondrial respiration (G) reflected by oxygen consumption rate (OCR) was measured by Seahorse in STARD3*^kd^* or STARD3*^NC^* for every 10 min for 80 min after treated with vehicle, 6% CSE or Chol/CSE combination, from which levels of basal respiration, proton leak, spare respiration capacity and ATP production were calculated (H). *p < .05; **p < .01. * represents the intragroup comparison, and # represents the intergroup comparison

CSE/cholesterol significantly reduced mitochondrial mass (Figure 5A) and MMP (Figure 5B,C) in HBEs, while levels of MMP in STARD3*^kd^* were higher than those in STARD3*^NC^*, which was observed in BEAS‐2B cells (Figure S6A–C). We measured the mitochondrial texture scores and morphology index using confocal microscopic imaging and performed the high content screening. The results illustrated that the short and fragmented mitochondria were partly recovered into long and tubular mitochondria in STARD3*^kd^* as compared with STARD3*^NC^* after CSE/cholesterol (Figure 5D). Levels of Filipin III intensity in mitochondria decreased (Figure 5E) and mitochondrial morphology increased (Figure 5F) in STARD3*^kd^*, respectively. The length/width ratio increased in STARD3*^kd^* after CSE/cholesterol (Figure S6D,E). Differences of mitochondrial ultrastructure were noticed between STARD3*^NC^* and STARD3*^kd^* with vehicle (Figure S7A,B), CSE (Figure S7C and S7D) or cholesterol (Figure S7E,F). Levels of basal respiration, proton leak, spare respiratory capacity and ATP production in HBEs with CSE were significantly lower than those with vehicle and became even lower in those with CSE/cholesterol (Figure 5G,H). Exogenous cholesterol did not exacerbate the respiration dysfunction in STARD3*^kd^*, while the levels of mitochondrial respiration in STARD3*^oe^* were lower than those in STARD3*^Ve^* (Figure S6F,G). The overexpression of STARD3 exacerbated the dysfunction of mitochondrial respiration induced by CSE. Lower levels of STARD3 alleviated the mitochondrial cholesterol overload and partially rescued MMP function, morphology and respiration.

**FIGURE 6:**
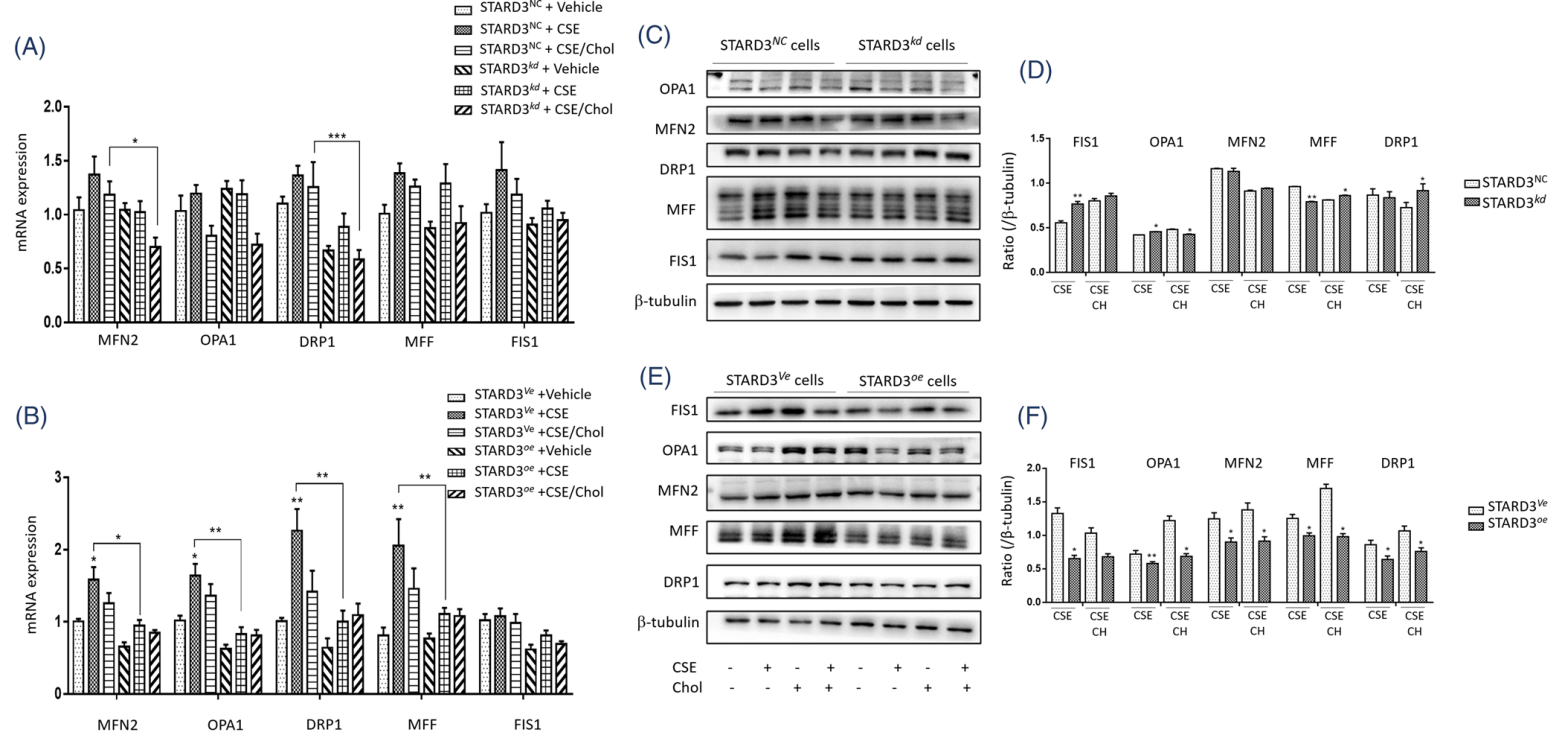
Roles of steroidogenic acute regulatory‐related lipid transfer domain‐3 (STARD3) in mitochondrial dynamics after cigarette smoke extract (CSE)/cholesterol stimulation. mRNA levels of mitofusin 1 and 2 (MFN1 and MFN2), optic atrophy 1 (OPA1), dynamin‐related protein 1 (DRP1), mitochondrial fission factor (MFF) and mitochondrial fission protein 1 (FIS1) were assayed in STARD3*^kd^* or STARD3*^NC^* after treated with vehicle, 6% CSE or Chol/CSE combination (A). The mRNA levels of MFN2, OPA1 DRP1, MFF and FIS1in STARD3*^oe^* or STARD3*^ve^* were analyzed by RT‐qPCR. (C) Western blot of MFN2, OPA1, DRP1, MFF and FIS1 in STARD3*^kd^* or STARD3*^NC^*. (D) The protein level from Western blot (C) analyzed with Image J. (E) Western blot of MFN2, OPA1, DRP1, MFF and FIS1 in STARD3*^oe^* or STARD3*^ve^*. (F) The protein level from Western blot (E) analyzed with Image J. *p < .05; **p < .01

We also evaluated the mitochondrial dynamics‐associated markers and found that mRNA expression of MFN2, OPA1, DRP1, MFF and FIS1 in HBEs with CSE were higher than those with vehicle or with CSE/cholesterol (Figure 6A). The expression of mitochondrial dynamics‐associated genes was lower in STARD3*^kd^*, as compared with STARD3*^NC^*. MFN2 expression was significantly lower in STARD3*^oe^* with CSE or CSE/cholesterol, as compared with STARD3*^Ve^* (Figure 6B). STARD3*^oe^* had lower expressions of DRP1 gene (Figure 6B) and protein (Figure 6E,F) than those of STARD3*^Ve^* after challenges with vehicle, CSE or CSE/cholesterol. Protein levels of FIS1 and OPA1 elevated in STARD3*^kd^* with CSE, while MFF and DRP1 elevated in STARD3*^kd^* with CSE/cholesterol, when compared between STARD3*^NC^* (Figure 6C,D). Levels of FIS1, OPA1, MFN2, MFF and DRP1 were down‐regulated in STARD3*^oe^* with CSE or CSE/cholesterol, as compared with STARD3*^Ve^* (Figure 6E,F).

### 2.6 MFN2 down‐regulation causes fatty acid oxidation and mitochondrial dysfunction

**FIGURE 7:**
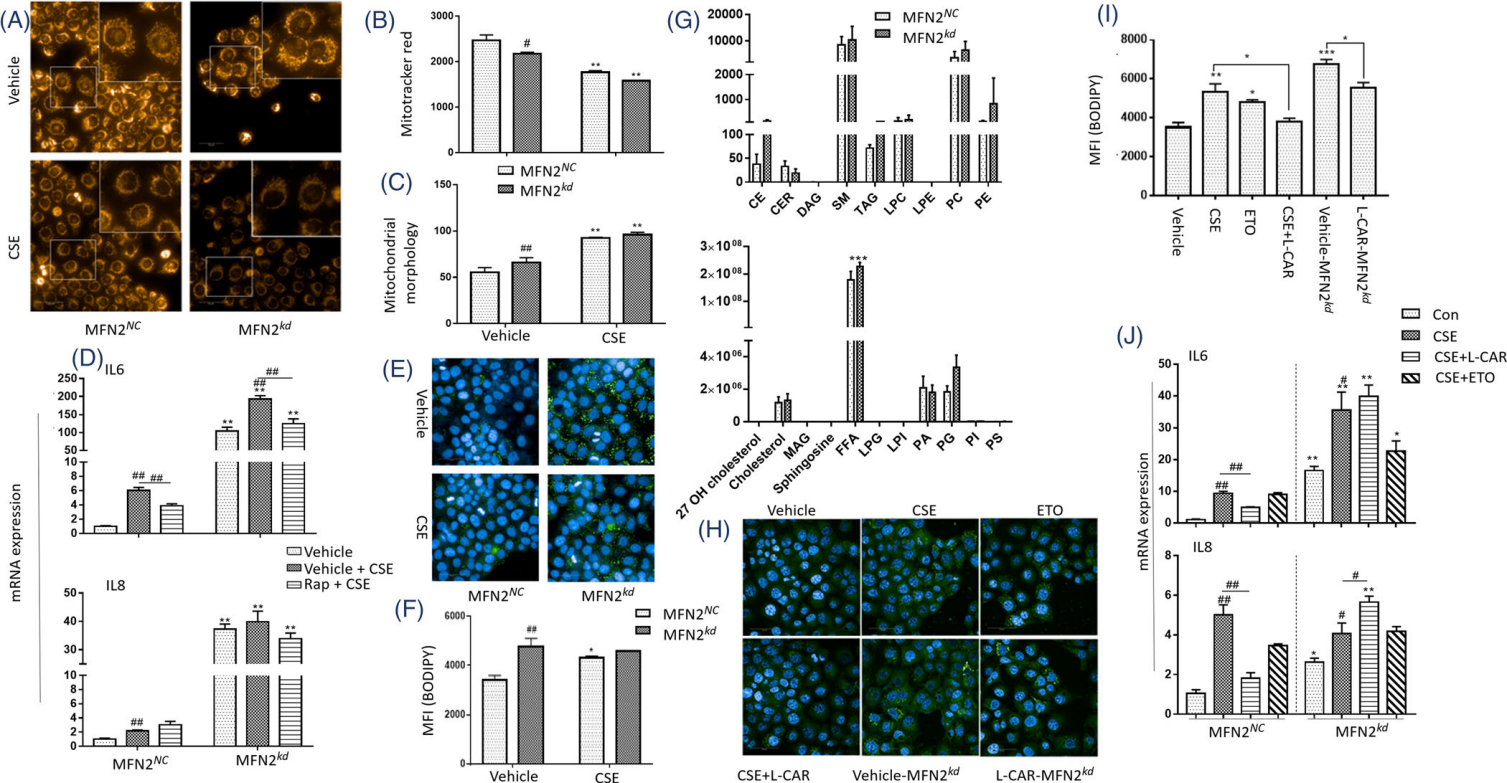
Down‐regulation of mitofusin 2 (MFN2) exacerbated mitochondrial dysfunction and cellular inflammation and led to the changes in lipid homeostasis. Human bronchial epithelial cells (HBEs) were stained with mitotracker red to represent mitochondrial membrane potential in MFN2 knockdown (MFN2*^kd^*) or negative control (MFN2*^NC^*) treated with 6% cigarette extract smoke (CSE) or vehicle (A). Mean fluorescence intensity (MFI) (B) and mitochondrial morphology scores (C) were calculated with Perkin‐Elmer Harmony image analysis software. The mRNA level of (D) interleukin (IL) 6 and IL8 by RT‐qPCR were analyzed in HBEs treated with 200 nM rapamycin (Rap) 2 h before 6% CSE stimulation. HBEs were stained with BODIPY 493/503 to show lipid droplets (LDs) and scanned by high content screening with 40X or 63X water object and confocal mode (E), and MFI (F) was analyzed by Perkin‐Elmer Harmony image analysis software. Lipidomics of MFN2*^kd^* and MFN2*^NC^* were analyzed, and the compounds, which had internal standard, were shown as quantitate data, and those, which did not have internal standard, were shown as original area data (G). LDs were stained by BODIPY 493/503 and scanned by high content screening with 40X or 63X water object and confocal mode (H), and MFI (I) was calculated by Perkin‐Elmer Harmony image analysis software in MFN2*^kd^* and MFN2*^NC^* treated with 5 μM etomoxir (ETO) or 50 μM L‐cartinine (L‐CAR) 2 h before 6% CSE stimulation. mRNA levels of IL6 and IL8 were assayed by RT‐qPCR in MFN2*^kd^* and MFN2*^NC^* treated with 5 μM etomoxir (ETO) or 50 μM L‐cartinine (L‐CAR) 2 h before 6% CSE stimulation (J). *p < .05; **p < .01. * represents the intragroup comparison, and # represents the intergroup comparison

**FIGURE 8:**
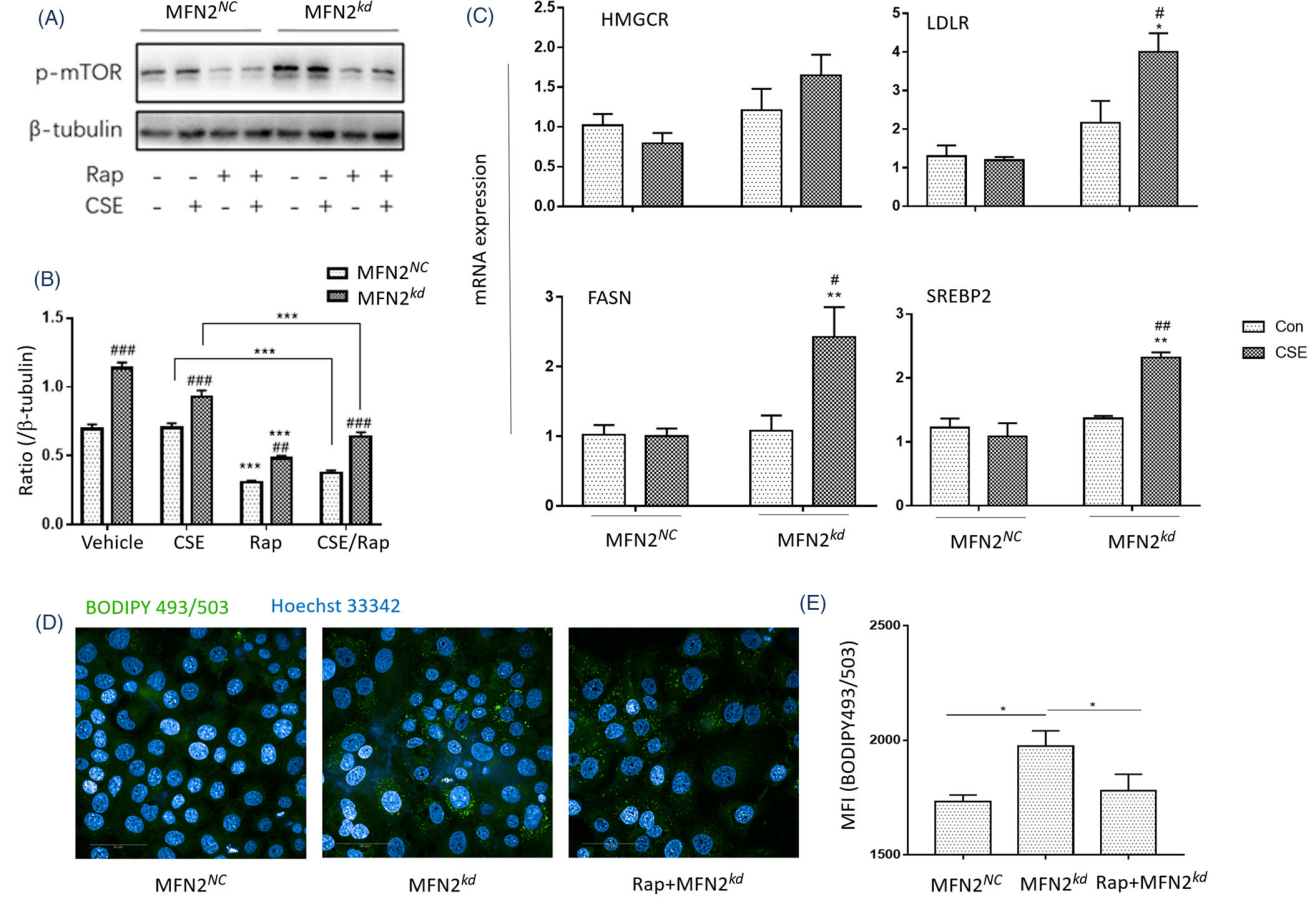
mTOR signalling pathway was activated after mitofusion (MFN)2 down‐regulation, which led to positive feedback in lipid accumulation and inflammation. mTOR phosphorylation in MFN2 knockdown (MFN2*^kd^*) or negative control (MFN2*^NC^*) stimulated with 6% cigarette smoke extract (CSE) ± 200 nM Rapamycin (Rap) was analyzed by Western blot (A), and the protein level (B) was calculated by Image J. mRNA level of 3‐hydroxy‐3‐methylglutaryl‐coenzyme A reductase (HMGCR), fatty acid synthase (FASN), low‐density lipoprotein receptor (LDLR) and sterol‐regulatory element binding protein 2 (SREBP2) in MFN2*^kd^* and MFN2*^NC^* stimulated with 6% CSE for 24 h were analyzed by RT‐qPCR (C). Lipid droplets (LDs) stained by BODIPY 493/503 in MFN2*^NC^* and MFN2*^kd^* stimulated with 6% CSE ± 200 nM Rap were scanned by Perkin‐Elmer Operetta high‐content screening (HCS) with 40X or 63X water object and confocal mode (D), and the mean fluorescence intensity (MFI) (E) was analyzed by HCS image analysis software. *p < .05; **p < .01. * represents the intragroup comparison, and # represents the intergroup comparison

MFN2 was upregulated in the lung tissues of CS/LPS‐treated mouse model and CSE‐stimulated HBEs, while downregulated by exogenous cholesterol supplement. We knocked down MFN2 (MFN2*^kd^*) and found the fragmentated mitochondria (Figure 7A), decreased MMP (Figure 7B), and altered mitochondrial morphology (Figure 7C) in MFN2*^kd^*. mRNA expression of IL‐6 and IL‐8 elevated in MFN2*^kd^* with CSE (Figure 7D), indicating that the downregulation of MFN2 may increase epithelial capacity of inflammatory reactions. We measured the lipid droplets (LDs) and found that MFN2*^kd^* had more LDs than MFN2*^NC^* and CSE increased LDs contents in MFN2*^NC^* (Figure 7E,F). The lipidomic measurements of MFN2*^NC^* and MFN2*^kd^* showed that downregulation of MFN2 elevated level of free fatty acid (FFA), cholesterol ester (CE) and triacylglycerol (TAG) (Figure 7G). The mitochondrial dysfunction leads to the disruption in fatty acid oxidation (FAO), and excessive FFA in cytoplasm turns into triacylglycerol storing in LDs. We applied etomoxir (ETO), an inhibitor of carnitine palmitoyl‐transferase 1α, for inhibiting FAO, and L‐carnitine (L‐CAR), an endogenous molecule for promoting FAO and interrupting the formation of LDs through the transport of long chain fatty acyl‐CoAs into the mitochondria. ETO and CSE increased LDs in MFN2*^NC^* (Figure 7H,I). L‐CAR reduced the production of pro‐inflammatory cytokines in CSE‐stimulated MFN2*^NC^* (Figure 7J), while L‐CAR aggravated the inflammatory response of MFN2*^kd^* with CSE. Levels of mTOR phosphorylation in MFN2*^kd^* were significantly higher than in MFN2*^NC^*, which was prevented by Rapamycin (Rap) (Figure 8A,B, *p* < .01, respectively). mRNA levels of lipid synthesis‐related genes, including SREBP2, HMGCR, FASN and LDL‐R, were higher in CSE‐stimulated MFN2*^kd^* (Figure 8C). Rap partially prevented the increase of LDs in MFN2*^kd^* (Figure 8D) and the production of IL‐6 in MFN2*^NC^* and MFN2*^kd^* (Figure 7D).

## 3 DISCUSSION

The abnormality of systemic lipidomic profiles is characterized and varies among lung diseases, including lung cancer, acute lung injury, pneumonia, pulmonary thrombosis and acute exacerbation of COPD.^22^, ^23^, ^24^ Lipidomic changes are associated with severities, durations, stages, clinical phenomes, subtypes and prognosis of lung disease. The circulating levels of lipid elements have been suggested as biomarkers for metabolism‐based diagnosis, monitors for therapeutic effects and lipid‐oriented mechanisms,^25^, ^26^, ^27^, ^28^ although there are still clinical and technical obstacles to be overcome. Metabolic disorders of palmitic or stearic acids in lung cancer or normal epithelial cells influenced cell proliferation through regulation of lipid‐associated genes.^29^ Of multiple lipid metabolisms and regulations, the dysfunction of cholesterol accumulation, trafficking and metabolism plays important roles in the development of lung chronic diseases, including diffuse parenchymal lung diseases, lung cancer and COPD.^30^, ^31^, ^32^ High level of cholesterol in plasma was found to predict complications, severities, stages and medication needs of patients with COPD. However, clinical studies demonstrated a conflicting therapeutic effect of cholesterol reduction by statins on clinical phenomes and outcomes of patients with COPD.^33^, ^34^, ^35^ It is still questioned whether the hypercholesterolemia can change lung epithelial cell sensitivity in response to external factors like smoking, accelerate epithelia‐driven local inflammation through hyper‐production of inflammatory mediators like cytokines, and contribute to metabolic disorders‐oriented organelle dysfunction like mitochondria. There is still a lack of direct evidence how external cholesterol induces the secondary accumulation of intra‐epithelial cholesterol and exacerbates the inflammation in the lung. The current study demonstrated the existence of hypercholesteremia in patients with COPD, evidenced by alternations of cholesterol‐related lipid genes in CSE‐treated epithelia and presented preclinical data to show that HCD worsened CS/LPS‐induced lung tissue inflammation and injury. Our data initially illustrated that the external cholesterol could change the inflammatory sensitivity of lung epithelial cells in response to smoking and indicated that the cholesterol transports may be associated with the production of ILs.

External cholesterol directly influences the intracellular haemostasis of cholesterol and inflammatory responses of airway epithelial cells through dysfunction of mitochondrial dynamics. We furthermore found that external cholesterol changed transcriptomic expression of mitochondria‐associated genes and caused the cholesterol accumulation within airway epithelial cells, accompanied by the down‐regulation of the SREBP2/LDL‐R pathway and catalyzation pathway of cholesterol biosynthesis. The cholesterol‐induced down‐regulation of SREBP2, HMGCR and LDLR expressions indicates that the negative feedback regulation exists, and the interaction between mitochondria and endoplasmic reticulum may be influenced in airway epithelial response to external cholesterol. Those factors play important roles in communication between endoplasmic reticulum and mitochondria responsible for cholesterol homeostasis, intra‐ and extra‐cellular microenvironment, metabolisms, signalling activities and cell–cell communication.^36^ The external cholesterol changes the sensitivity of airway epithelial cells probably through down‐regulating mitochondrial function, for example, mitochondrial respiration, mitochondrial membrane, several metabolic pathways and dynamics, or through the impairment of mitochondrial OXPHOS and respiratory super complexes, like in other cells.^37^ High cholesterol level decreased limited mitochondrial ability to adapt to CSE exposure as evidenced from increase in mitochondrial mass that is not accompanied by neither stabilization of MMP, nor increased respiration. Heightened dysregulation of fusion/fission process, especially the opposite effects of cholesterol to CSE on MFN2 regulation, could account for cholesterol effects on adaptive responses.

STARD3 contributed to the regulation of cholesterol‐induced IL production from airway epithelial cells. It was evidenced by the fact that cholesterol down‐regulated the expression of STARD3, changed epithelial sensitivity to smoking and lost/increased effects of IL production when STARD3 was down‐ or over‐regulated. The cholesterol is transported from intracellular organelle pools to mitochondrial membrane through the interaction between those transfer proteins with the mitochondrial protein complex for biological functions of mitochondria, including biogenesis, membrane maintenance and synthesis of steroids, oxysterols and hepatic bile acids.^36^, ^38^, ^39^ Our data showed that STARD3‐down‐regulated epithelial cells had low capacities of inflammatory mediator production and low sensitivity in response to smoking. Cholesterol‐modulated airway epithelial sensitivity was partially dependent upon STARD3‐regulated mitochondrial function. Our results demonstrated that the down‐regulation of STARD3 led to epithelial mitochondrial dysfunction and less sensitivity to challenges, probably through re‐distribution of intracellular cholesterol, hypoproduction of mitochondrial reactive oxygen species, or inactivation of peroxisome proliferator‐activated receptor‐γ and CCAAT/enhancer binding protein.^12^ It was evidenced by the finding that the down‐regulation of STARD3 decreased the mitochondrial cholesterol level and partly reversed the mitochondrial function including MMP and morphology impaired by CSE and cholesterol. Those mitochondrial dysfunctions were associated with the disorder of oxidative stress and phosphorylation^40^ and found to contribute to the damage of airway epithelial cells in smoking model.^41^ The airway epithelial sensitivity to CSE was altered by adding cholesterol, when mitochondrial dynamics and function changed more obviously in the condition of STARD3 over‐expression. On the other hand, CSE stimulation could cause alternations of mitochondrial fusion and fission genes and proteins when STARD3 gene was manipulated, indicating that CSE‐induced mitochondrial dysfunction in OXPHOS may have other independent mechanisms of STARD3 and intracellular cholesterol homeostasis.

The mitofusin‐associated regulations contributed to CSE‐altered mitochondrial fusion and subcellular cholesterol trafficking in airway epithelial cells. Disorders of mitochondrial dynamics were noticed in lung epithelial cells and proposed to be the part of pathogenesis of various lung diseases including COPD.^14^, ^42^ CSE increased mitochondrial fission and reduced mitochondrial fusion in CSE‐induced pulmonary endothelial injury and short OPA1 isoforms increased in COPD subjects.^16^, ^43^ Of fusion and fission proteins, we found that MFN2 expressed mostly in human airway epithelia, CSE increased MFN2 expression, and adding cholesterol could down‐regulate CSE‐induced over‐expression of MFN2. The deletion of MFN2 reduced the production of surfactant lipids, synthesis of phospholipids and cholesterol and activity of fatty acid synthase, leading to the exacerbation of lung injury and high morbidity and mortality.^44^ It is possible that the up‐regulation of MFN2 may be a compensatory and protective effect in response to CSE, while external cholesterol inhibited such defensive sensitivity and increased airway epithelial susceptibility of mitochondrial dynamics to CSE. Recent study showed that STARD3 overexpression down‐regulated MFN2 independently of mitochondrial morphology. ^45^ Our data furthermore indicate that STARD3 overexpression seemed to contribute more to the regulation of mitochondrial dynamics than STARD3 down‐regulation did. Chronic exposure to CS increases STARD3 levels and contributes to inflammation. This increase of STARD3 levels is diminished by high cholesterol. Nevertheless, pro‐inflammatory signalling is the most prominent in cells exposed to the combination of CSE and high cholesterol. Therefore, high cholesterol increases the levels of pro‐inflammatory cytokines probably via additional to STARD3 pathway. STARD3 overexpression altered mitochondrial dynamics by down‐regulating the dynamics‐associated proteins in response to challenges, leading to strong accumulation of damaged mitochondria, as reported in the aging.^46^

Down‐regulation of MFN2 increased airway epithelial sensitivity and production of ILs, especially IL‐6, in response to smoking, at least partially by activating the mTOR phosphorylation. The disruption of mitochondrial dynamics could impair the lipid metabolism to activate the mTOR pathway and affect the cellular lipid homeostasis.^47^, ^48^, ^49^ We found that CSE stimulation or MFN2 down‐regulation increased LDs, leading to the impaired transfer of fatty acid between LDs and mitochondria and the abnormal lipidomic profiles of airway epithelial cells. It is possible that the impaired FAO during the development of mitochondrial dysfunction may result in the retention of free fatty acids in cytoplasm and synthesis of TAG, which is the main compound in LDs.^50^, ^51^, ^52^ It was supported by the findings that ETO inhibited the fatty acid oxidation and caused LDs accumulation, while L‐CAR promoted the process and down‐regulated the LDs accumulation induced by CSE or MFN2 knockdown.

With rapid development of biotechnology, the interaction and intercommunication among those genes, proteins and cells will be furthermore clarified and understood. For example, spatial transcriptomics has been recently applied to identify cell–cell communication, define in situ expression of transcriptomic profiles and provide new insights of understanding molecular mechanisms in lung cancers, although there are still challenges to be overcome.^53^ The molecular mechanisms by which those multi‐factors and multi‐cells interact need to be furthermore defined. It should be clarified whether the mitochondrial dysfunction is a center to regulate airway epithelial sensitivity in response to pathogens or pollutions, whether the re‐distribution of intracellular cholesterol among organelles contributes to the inflammation, and whether cholesterol or cholesterol‐driven metabolites directly deteriorate epithelial capacities of inflammatory regulations. The accumulation of cholesterol and up‐regulated expression of cholesterol synthesis‐related genes in liver were proposed as one of potential reasons of hypercholesterolemia in experimental smoking.^54^ Although the hypercholesterolemia has been reported by several recent studies on COPD, the limited sample size and age/sex‐related bias in our clinical data might affect the results in our study, and further clinical investigation is necessary. CSE induced hyperproduction of pro‐inflammatory cytokines from the airway epithelia probably by CSE‐specific receptors and intracellular signalling pathway^20^, ^55^ and inhibited cell proliferation by CSE‐associated cytotoxicity or induction of cell apoptosis.^56^, ^57^, ^58^ The mitochondrial dysfunction could induce severe inflammation by various factors, including the activation of the inflammasome or STING activation, although the exact mechanisms should be explored. Cholesterol can turn into 7α, 25‐dihydroxycholesterol as the main ligand to bind Epstein–Barr virus‐induced gene 2 in immune cells, accelerating more inflammatory cells into lung tissues.^31^ Our data showed that high levels of cholesterol occurred in animal models induced by a 4‐week exposure of LPS and CSE and correlated with lung severe inflammation and structural damage, more like the acute exacerbation of COPD, although cholesterol dynamics and biophysics in the long‐term models with emphysema or biology models with the clear targeting are more expected. HBE cells retained differentiated epithelial morphology and functions in the liquid culture system,^59^ while the study on primary epithelial cells cultured in the air‐liquid interface system will be considered for further validation.

In conclusion, the cholesterol overload was associated with COPD and airway epithelia‐driven inflammation, evidenced by hypercholesterolemia in patients with COPD and smoking model, alteration of lipid metabolism‐associated genes in CSE‐induced airway epithelia and production of ILs. External cholesterol altered airway epithelial sensitivity to inflammation in response to CSE, through intracellular transport and accumulation of cholesterol, activities of lipid transport regulators, regulation of STARD3‐MFN2 pathways and mitochondrial dysfunction. Down‐regulation of MFN2 increased airway epithelial sensitivity and production of ILs after smoking, at least partially by activating the mTOR phosphorylation. Thus, our data provide new insights for understanding molecular mechanisms of cholesterol‐altered airway epithelial sensitivity and for developing diagnostic and therapeutic multi‐target panel to improve the patient prognosis.

## 4 METHODS

### 4.1 Patient population

The present study was approved by the Ethical Evaluation Committee of Zhongshan Hospital (B2019‐197(2)) and designed using a case–control approach. Informed consent for lipids analysis was given by patients. The study involved 22 patients with severe COPD, which was diagnosed as described previously.^60^ Patients were recruited between October 2019 and March 2020 with the medical history including admittance to the hospital due to acute exacerbation. Nineteen healthy volunteers were enrolled at Zhongshan Hospital. Plasma was harvested from healthy volunteers and patients with severe COPD on day one of admission. Details of patient population are listed in Table S1.

### 4.2 Cell culture, agents, transfection and RNA interference

HBE cells were derived from normal bronchus in a 1‐year‐old male heart‐lung transplant patient, immortalized cell lines, which were first extracted from bronchus.^59^ HBE cells were cultured in RPMI 1640 (SH30809.01, Hyclone, Logan, UT, USA) with 10% fetal bovine serum (#10099141, Gibco, ThermoFisher, MA, USA). BEAS‐2B cells, another normal epithelial cell line, were purchased from ATCC (CRL‐9609) and cultured in DMEM (SH30243.01, Hyclone, Logan, UT, USA). T0901317 (HY‐10626), GW3965 (HY‐10627), rapamycin and atorvastatin (HY‐B0589) were purchased from MedChemExpress, NJ, USA. Transfection of RNA interference was conducted by Lipofectamine 2000 (#11668019, ThermoFisher Scientific, MA, USA). The sequencing is listed as below:

siRNA‐STARD3‐sense: CCUGGUUCCUUGACUUCAATT;

siRNA‐STARD3‐nonsense: UUGAAGUCAAGGAACCAGGTT;

siRNA‐MFN2‐sense: CCAUGAGGCCUUUCUCCUUTT;

siRNA‐MFN2‐nonsense: AAGGAGAAAGGCCUCAUGGTT.

Transfection of STARD3 overexpression plasmid (Genechem, Shanghai, China) was conducted by Lipofectamine 3000 (L3000008, ThermoFisher Scientific, MA, USA). The knock down efficiency was verified after 48 h. Targeted‐STARD3 shRNA lentiviral vector was constructed in Genechem, Shanghai, China, and transfected into HBE cells according to the manufacturer's protocol. Stable HBE cells with lower STARD3 expression were verified by RT‐qPCR as well as Western blot. All in vitro experiments were performed and triplicated, and there were six per group each time.

### 4.3 CSE preparation

Briefly, one cigarette was combusted through a modified 50‐ml syringe apparatus into 5‐ml RPMI 1640. The quality control of 100% CSE was performed by measuring the optical density at 320λ wavelength and a value of 1.158.^61^

### 4.4 Cholesterol preparation

Cholesterol was purchased from Sigma‐Aldrich, Louis, USA (#C3045) and was dissolved in 100% ethanol at 65℃. Then, the liquid was added to methyl‐β‐cyclodextrin (HY‐101461, MedChemExpress, NJ, USA) at a concentration of 5 mM cholesterol to 42 mg/ml methyl‐β‐cyclodextrin.^62^

### 4.5 Cholesterol staining and high‐content screening

Cholesterol was stained by Filipin III in cells fixed with paraformaldehyde. For the observation on colocalization of cholesterol and mitochondria, cells were incubated with mitotracker deep red (25 nM) for 30 min before fixation. Fixed cells were incubated with 50 μg/ml filipin III (SAE0088, Sigma, MO, USA) at room temperature for 1 h. The emission/excitation of filipin III were 340–380/385‐470 nm. Cells were screened with Perkin‐Elmer high‐content automatic imaging system (40× air or 63× water objective) in confocal mode, and the results were analyzed by Perkin‐Elmer Harmony image analysis software.

### 4.6 Analysis of mitochondrial function

The mitochondrial function was evaluated, including mitochondrial mass, MMP, morphology, respiration and mitochondrial dynamics. For mitochondrial MMP analysis, live cells were stained with mitotracker green (C1048, Beyotime, Shanghai, China, 100 nM) and mitotracker red CMXRos (C1049, Beyotime, Shanghai, China, 25 nM) in 1640 Medium at 37℃ for 30 min and evaluated by flow cytometry or high‐content screening. For the analysis of mitochondrial morphology, the texture of mitochondria was extracted using Perkin‐Elmer Operetta high‐content automatic imaging system. Texture parameters of the mitochondria—SER features (mathematical basis is Gaussian derivative filter) including eight patterns (Spot, Edge, Ridge, Saddle, Valley Hole, Bright, Dark) at 1px scale with Kernel normalization were determined to measure mitochondrial morphology.^63^, ^64^ The scores of SER Ridge were calculated to quantify the mitochondrial morphology. In transmission electron microscopy, cells were collected ang fixed by 2.5% glutaraldehyde in 0.1 M sodium cacodylate pH 7.4. ImageJ was used to measure the ratio of length/width of the mitochondria as recently reported.^65^ For the analysis of mitochondrial respiration, cell mitochondrial stress test kits (103015‐100, Agilent Technologies, CA, USA) were used. OCR was measured using a Seahorse XFe96 Extracellular Flux Analyzer (Agilent Technologies, CA, USA).^66^ Briefly, HBEs were plated on 96‐well plates at 1*10^5^ cells/well in RPMI 1640 including 10 mM glucose, 2 mM l‐glutamine and 1 mM sodium pyruvate. ECAR and OCR were detected at basal level and after injections of 2 μM oligomycin, 1 μM FCCP and 0.5 μM rotenone/antimycin mix. Cell number was averaged per condition, and OCR was normalized to these values. Basal respiration, proton leak, spare respiratory capacity and ATP production were calculated. For the analysis of mitochondrial dynamics, RT‐qPCR and Western blot were used to evaluate MFN2, OPA1 as fusion markers and DRP1, MFF and FIS1 as fission markers.

### 4.7 LDs staining

Cells were stained with BODIPY 493/503 (GC42959, GLPBIO, CA, USA, 10 μM) for 60 min to visualize LDs in fixed cells. HCS (63X water object, confocal mode) was used to scan the cells and calculate the mean fluorescence intensity.

### 4.8 GC‐MS/MS metabolomics to evaluate cholesterol level

Plasma from healthy volunteers and COPD patients or BALF from mouse models were obtained. Regarding derivatization, methoxyamine with N‐methyl‐N(trimethylsilyl)trifluoroacetamide (MSTFA) was applied.^67^ Briefly, 100 μl of fresh plasma sample was treated with 400 μl of cold methanol‐water (4:1 v/v, 5ug/ml tridecanoic acid was included). The mixture was vortexed, shacked at 37°C, 1200 rpm for 30 min and then centrifuged. The supernatant was evaporated under a stream of nitrogen gas. Methoxyamination of samples was carried out with 50 μl MeOX (20 mg/ml) in pyridine. Each sample was sonicated for 1 min and shaked at 30°C, 1200 rpm for 90 min, trimethylsilylated in 40 μl MSTFA and shook at 37°C, 1200 rpm for 30 min. The supernatant was clarified by centrifugation at 12000 rpm for 5 min and transferred to a vial for GC‐MS/MS analysis. The Agilent Fiehn GC/MS Metabolomics RTL Library (version 2013) was employed for metabolite identifications. Tridecanoic acid was added as an internal standard. The formula: standard sample peak area/standard sample concentration = sample peak area/sample concentration, was used to calculate the metabolites content in the sample.^68^

### 4.9 Quantitative RT–PCR

TRIzol (#15596026, Invitrogen, CA, USA) was used to extract total RNA. Then, mRNA was reverse‐transcribed to cDNA by PrimeScript RT Master Mix (RR036A, Takara, Japan). The real‐time PCR was performed by TB Green Premix Ex Taq (RR420A, Takara, Japan) with primers listed in Table S4.

### 4.10 Western Blot

Ice‐cold lysis buffer (with protease inhibitors) was used to lyse cells. After centrifugation, total proteins in the supernatants were quantified using BCA kit (P0010, Beyotime, Shanghai, China). Note that 30‐μg protein was loaded per lane in 10% SDS‐PAGE and electrophoresed on a PVDF membrane for detection with antibodies. Primary antibodies are as follows: β‐tubulin (#2128, 1:1000, Cell Signalling Technology, MA, USA); β‐actin (#4970, 1:1000, Cell Signalling Technology, MA, USA), p‐mTOR (phosphor S2448, 1:1000, Cell Signalling Technology, MA, USA), MFN2 (ab124773, 1:1000, Abcam, Cambridge, UK), DRP1 (ab184247, 1:1000, Abcam, Cambridge, UK), OPA1 (ab157457, 1:1000, Abcam, Cambridge, UK), MFF (#17090‐1‐AP, 1: 5000, Proteintech, IL, USA) and FIS1 (#10956‐1‐AP, 1:1000, Proteintech, IL, USA). Secondary antibodies were HRP linked anti‐mouse/rabbit IgG (1:2000, #7076/#7074, Cell Signalling Technology, MA, USA). The bound antibodies were visualized with ECL detection (#32106, ThermoFisher Scientific, MA, USA). The gray values were calculated by Image J (NIH, USA).

### 4.11 Animal models

Wild‐type C57BL/6J mice (6–8 weeks old) were purchased from Shanghai Jiesijie Animal Experiment Co., Ltd. (Shanghai, China) and housed in a temperature‐ and humidity‐controlled room in Zhongshan Hospital Animal Experiment Center. CSE/LPS‐induced mouse models were induced as previously reported.^69^, ^70^ Briefly, mice were intraperitoneally injected with 200 μl 2.5% avertin (T48402, Sigma, MO, USA) for anesthesia and intratracheally instilled with 25 μg LPS (L2880, Sigma, USA) in 50 μL saline on days 0 and 15. On days 1∼14 and 16∼29, the mice were exposed to CS (1 h/time, 20 cigarettes/day) in a closed organic glass box (80 × 60 × 50 cm). Each cigarette contains 10‐mg tar, 0.8‐mg nicotine and 12‐mg carbon monoxide. HCD was supplied by Research Diets (D12109, Research Diets, NJ, USA).^71^ Mice were sacrificed at day 30, and the blood, BALF and lung tissue were collected for further analysis. The mouse lung tissues were processed for protein and/or RNA measurements. For RNA, 50‐mg tissues in liquid nitrogen were added 1‐ml TRIzol and homogenized by tissue grinder, followed by RNA extraction using chloroform and isopropanol. For protein, 50‐mg tissues in liquid nitrogen were added 300‐μl ice‐cold lysis buffer plus protease inhibitors and homogenized by tissue grinder, followed by the procedures mentioned in Western blot section.

### 4.12 HBE RNA sequencing

Cellular RNA was extracted by TRIZOL, and the integrity was assessed using the RNA Nano 6000 Assay Kit of the Bioanalyzer 2100 system (Agilent Technologies, CA, USA). AMPure XP system (Beckman Coulter, Beverly, USA) was used for purification. Illumina Novaseq platform was used for sequencing. Data were aligned to the reference genome using Hisat2 v2.0.5. FeatureCounts v1.5.0‐p3 was used to count the reads numbers mapped to each gene, and Fragments Per kilobase Million (FPKM) of each gene was calculated. DEGs were analyzed by DESeq2 R package (1.20.0). Gene ontology enrichment analysis and KEGG pathways were implemented by DAVID Bioinformatics Resources 6.8. 1. Differential mitochondria‐associated genes from HBE RNA sequencing were filtered by Human MitoCarta 3.0 datasets.

### 4.13 mRNA expression of STARD family in RNAseq data

RNAseq library preparation and sequencing were processed following the instruction in the section ‘HBE bulk RNA sequencing’. RNAseq analysis was performed from three independent replicates for each condition or group. Then, we obtained the RNAseq BAM files in the RNAseq database. Gene expression analysis of STARD family was performed in six independent experiments of different conditions. For heatmap, RNAseq reads were normalized (RPKM) for all conditions, and variations were scored as a Log ratio between different conditions or groups. One‐way ANOVA test was used to test the significance of differences between conditions by R language and Prism 8.0.

### 4.14 Analysis of intracellular lipidomics

Cultured cells were collected and normalized to 2*10^6 cells/sample, and centrifuged. Cell pellets were added with 0.9 ml double distilled water and disrupted by repeated freeze‐threw cycles for three times. Lipid internal standard mixture was added. Lipid extraction was performed using a modified Bligh and Dyer procedure, as described preciously.^72^ The extracts were dissolved by 200 μl chloroform/methanol (1:2, v/v, including 10mM NH_4_Ac) and stored at −20℃. We used normal phase liquid chromatography and coupled triple‐quadrupole mass spectrometer (QTRAP 5500, AB SCIEX, USA) to detect the lipid by positive and negative electrospray ionization mode. Q‐Trap in the multiple reaction monitoring mode operation was applied.^22^ Lipidomics data were analyzed by MultiQuant software (AB SCIEX, USA).

### 4.15 Statistical analysis

All quantitative data were calculated using GraphPad Prism Version 7.0 (Graph Pad Software, CA, USA) and presented as mean ± standard error of the mean. An unpaired two‐tailed Student's t‐test or ANOVA test was used to calculate the *p* values according to the different data types. *p <* .05 was considered significant.

## AUTHOR CONTRIBUTIONS

LYL contributed to study designs, experimental performance, data analysis, and manuscript preparation; YFL and XQL to RNA seq data analysis and result discussion; NNZ to experimental preparation; and XDW, YLS and YTG to the concept and design of the work as project leaders.

## CONFLICT OF INTEREST

The authors have no conflict of interest to disclose.

## FUNDING INFORMATION

National Nature Science Foundation of China, Grant Numbers: 81873409 and 12026608

## Supporting information

Figure S1 The mRNA level of 384 lipid metabolism‐related genes analyzed in human bronchial epithelial cells (HBEs) stimulated with cigarette smoke extract (CSE). The experimental procedure and volcano plots of all the genes were illustrated (A), and heatmaps (B) of 67 differentially expressed genes (C) were shown with their *p* value and fold change.
Figure S2 The viability data of human bronchial epithelial cells (HBEs) treated with different concentration of cigarette smoke extract (CSE) analyzed by CCK‐8. *p < .05, compared with group ‘Control’.
Figure S3 The RNA‐Seq data of human bronchial epithelial cells (HBEs) stimulated with 100 μM cholesterol for 6/24 h, 6% cigarette smoke extract (CSE) for 24 h and 6% CSE+100 μM cholesterol for 24 h. *n* = 3/group. PCA analysis (A) and Venn analysis (B) were done, and top 50 differentially expressed genes (DEGs) were clustered and showed in Heatmap (C). Two hundred forty‐two mitochondrial DEGs were filtered by Mitocarta3.0 in CSE‐stimulated HBE with vehicle or cholesterol, and top 50 were showed in Heatmap (D). Protein‐protein interaction analysis (E) was conducted in the top 50 mitochondrial DEGs.
Figure S4 ECAR by Seahorse every 10 min for 80 min in human bronchial epithelial cells (HBEs) treated with vehicle, 6% cigarette smoke extract (CSE), 100 μM cholesterol or combination (CSE/Chol).
Figure S5 Electron microscope of human bronchial epithelial cells (HBEs) stimulated with vehicle (A) and 6% cigarette smoke extract (CSE) (B) or combination (CSE/Chol) (C) for 24 h. The transcriptional levels of mitochondrial dynamics‐related genes including mitofusin 1/2 (MFN1/2), optic atrophy 1 (OPA1), dynamin‐related protein 1 (DRP1), mitochondrial fission factor (MFF), mitochondrial fission protein 1 (FIS1) in HBE stimulated with vehicle, 6% CSE, 100 μM cholesterol or CSE/Chol were analyzed from RNA‐Seq data (D). The mRNA levels of IL6 (E), IL8 (F) were detected by RT‐qPCR in STARD3 knockdown (STARD3*^kd^*) or negative control (STARD3*^NC^*) BEAS‐2B cells stimulated with vehicle, 6% CSE, 100 μM cholesterol or CSE/Chol. *p < .05; **p < .01. * represents the intragroup comparison, # represents the intergroup comparison.
Figure S6 Mitochondrial mass by mitotracker green staining (A), mitochondrial membrane potential by mitotracker red staining (B) and standardized mitochondrial membrane potential by mitotracker red/green (C) were measured by flow cytometry in 6% cigarette smoke extract (CSE)‐stimulated BEAS‐2B cells with STARD3 knockdown (STARD3*^kd^*) or negative control (STARD*^NC^*). The morphology of mitochondria was observed by electron microscope (D) in STARD3*^kd^* and STARD*^NC^* treated with CSE/Chol, and the ratio of length/width (D) in each mitochondrial was calculated by Image J. Oxygen consumption rate (OCR) (F) was measured for every 10 min for 80 min by Seahorse in STARD3*^oe^* or STARD3*^Ve^* treated with vehicle or 6% CSE, from which levels of basal respiration, proton leak, spare respiration capacity and ATP production were calculated (G). *p < .05; **p < .01. * represents the intragroup comparison, and # represents the intergroup comparison.
Figure S7 The ultrastructure of mitochondria in STARD3*^NC^* (A), STARD3*^kd^* (B), STARD3*^NC^* +6% cigarette smoke extract (CSE) (C), STARD3*^kd^* +6% CSE(D), STARD3*^NC^* +100 μM cholesterol (E) and STARD3*^kd^* +100 μM cholesterol (F) were also scanned by electron microscope. The magnification of each image was 3000X, 5000X, 10000X and 20000X from the top to the end.
