# Supplementary material for: Regulatory roles of external cholesterol in human airway epithelial mitochondrial function through STARD3 signalling
Source: Clin Transl Med. 2022 Jun 9;12(6):e902. doi: 10.1002/ctm2.902 (PMC9178408; doi:10.1002/ctm2.902)
Supplement: Supplementary file 1 — Figure S1 The mRNA level of 384 lipid metabolism‐related genes analyzed in human bronchial epithelial cells (HBEs) stimulated with cigarette smoke extract (CSE). The experimental procedure and volcano plots of all the genes were illustrated (A), and heatmaps (B) of 67 differentially expressed genes (C) were shown with their p value and fold change. Figure S2 The viability data of human bronchial epithelial cells (HBEs) treated with different concentration of cigarette smoke extract (CSE) analyzed by CCK‐8. *p < .05, compared with group ‘Control’. Figure S3 The RNA‐Seq data of human bronchial epithelial cells (HBEs) stimulated with 100 μM cholesterol for 6/24 h, 6% cigarette smoke extract (CSE) for 24 h and 6% CSE+100 μM cholesterol for 24 h. n = 3/group. PCA analysis (A) and Venn analysis (B) were done, and top 50 differentially expressed genes (DEGs) were clustered and showed in Heatmap (C). Two hundred forty‐two mitochondrial DEGs were filtered by Mitocarta3.0 in CSE‐stimulated HBE with vehicle or cholesterol, and top 50 were showed in Heatmap (D). Protein‐protein interaction analysis (E) was conducted in the top 50 mitochondrial DEGs. Figure S4 ECAR by Seahorse every 10 min for 80 min in human bronchial epithelial cells (HBEs) treated with vehicle, 6% cigarette smoke extract (CSE), 100 μM cholesterol or combination (CSE/Chol). Figure S5 Electron microscope of human bronchial epithelial cells (HBEs) stimulated with vehicle (A) and 6% cigarette smoke extract (CSE) (B) or combination (CSE/Chol) (C) for 24 h. The transcriptional levels of mitochondrial dynamics‐related genes including mitofusin 1/2 (MFN1/2), optic atrophy 1 (OPA1), dynamin‐related protein 1 (DRP1), mitochondrial fission factor (MFF), mitochondrial fission protein 1 (FIS1) in HBE stimulated with vehicle, 6% CSE, 100 μM cholesterol or CSE/Chol were analyzed from RNA‐Seq data (D). The mRNA levels of IL6 (E), IL8 (F) were detected by RT‐qPCR in STARD3 knockdown (STARD3 kd ) or negative control (STARD3 NC [file CTM2-12-e902-s001.docx]

**Supplementary data**


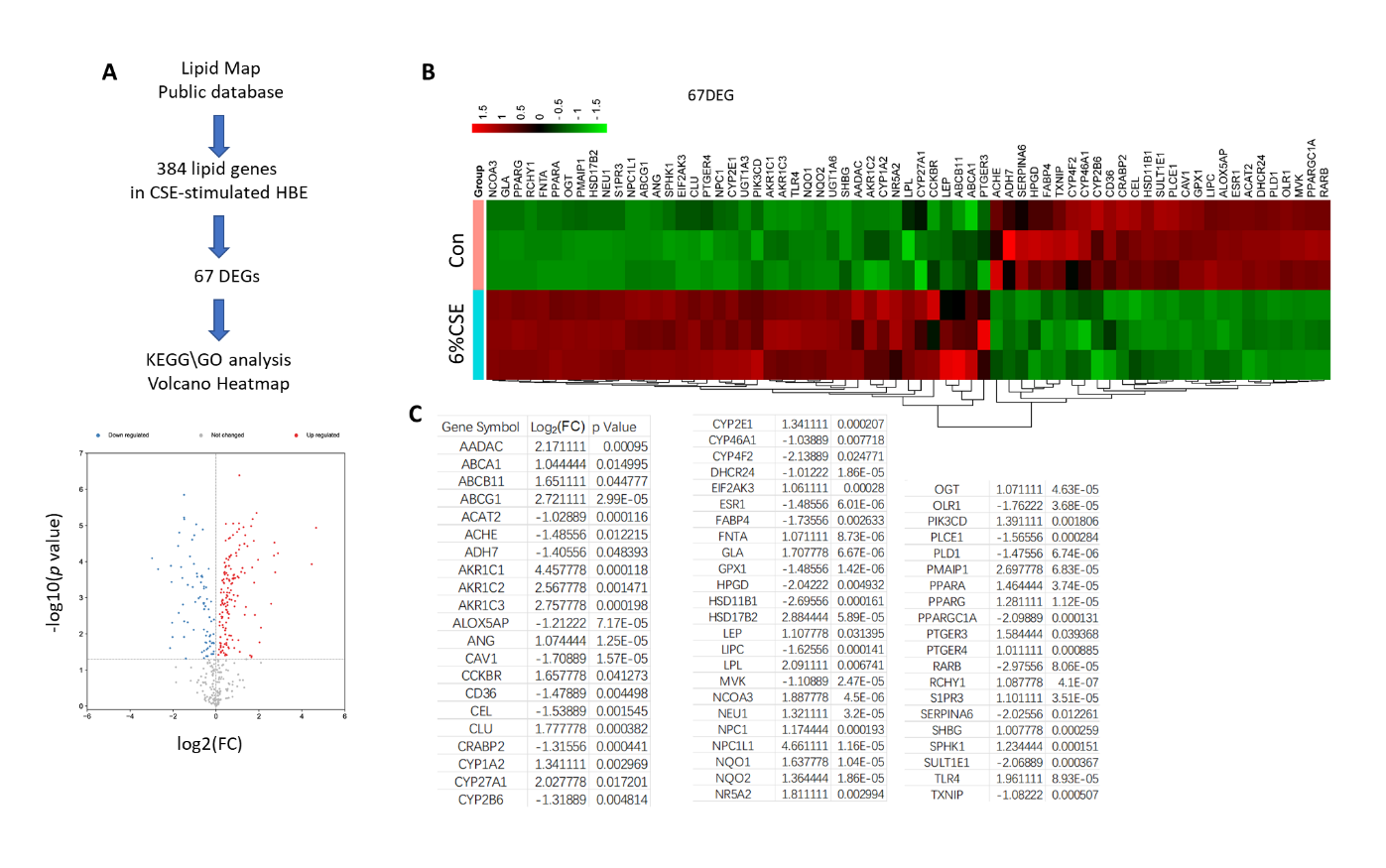


**Figure S1.** The mRNA level of 384 lipid metabolism-related genes analyzed in human bronchial epithelial cells (HBEs) stimulated with cigarette smoke extract (CSE). The experimental procedure and Volcano plots of all the genes were illustrated (A) and heatmap (B) of 67 differentially expressed genes (C) were shown with their p value and fold change.





**Figure S2** The viability data of human bronchial epithelial cells (HBE) treated with different concentration of cigarette smoke extract (CSE) analyzed by CCK-8. **p*<0.05, compared with group ‘Control’.


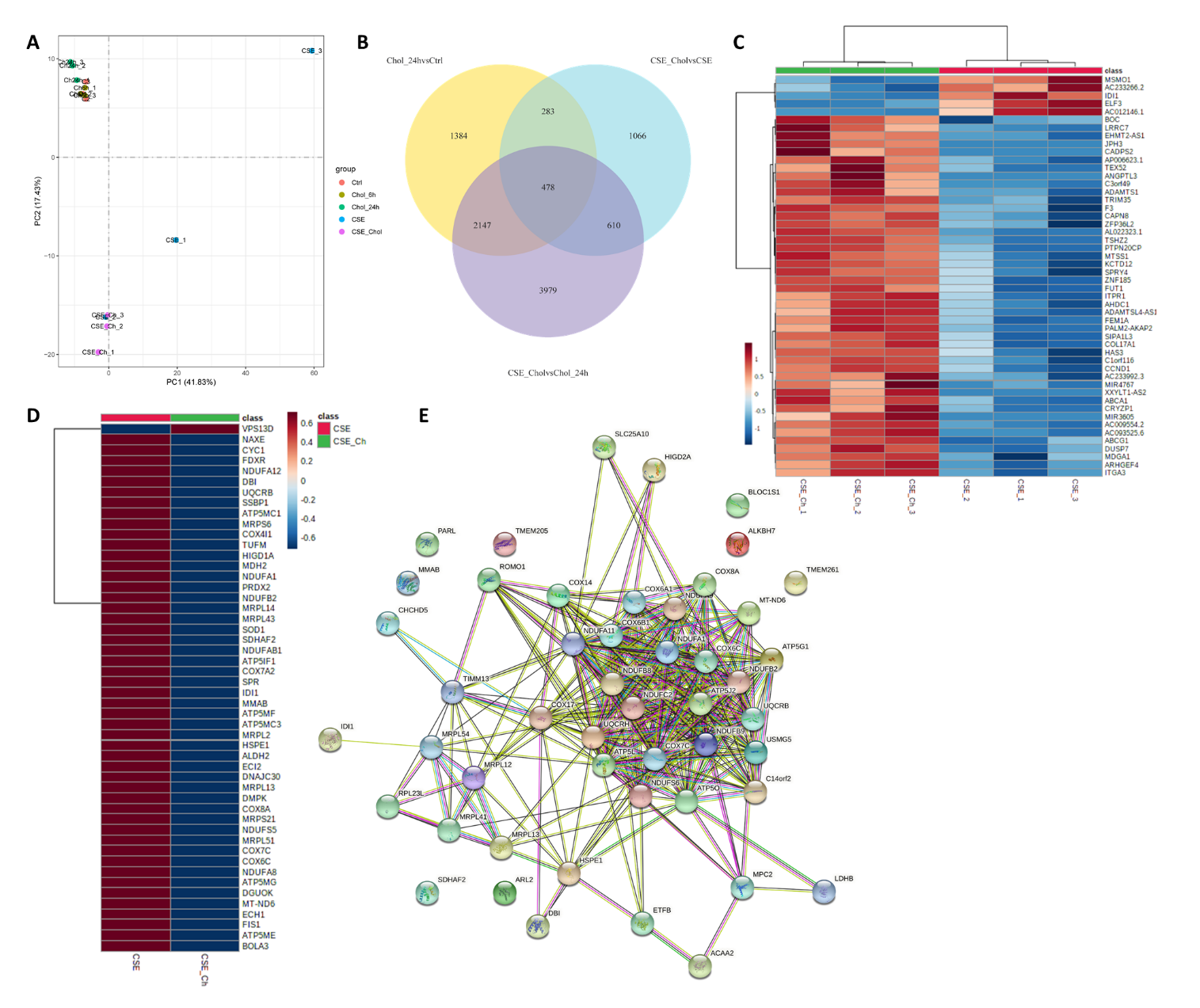
**Figure S3.** The RNA-Seq data of human bronchial epithelial cells (HBEs) stimulated with 100μM cholesterol for 6/24 hours, 6% cigarette smoke extract (CSE) for 24 hours and 6% CSE+100μM cholesterol for 24 hours. n=3/group. PCA analysis (A) and Venn analysis (B) were done, and top 50 differentially expressed genes (DEGs) were clustered and showed in Heatmap (C). 242 mitochondrial DEGs were filtered by Mitocarta3.0 in CSE-stimulated HBEs with vehicle or cholesterol and top 50 were showed in Heatmap (D). Protein-protein interaction analysis (E) was conducted in the top 50 mitochondrial DEGs.

**Figure S**
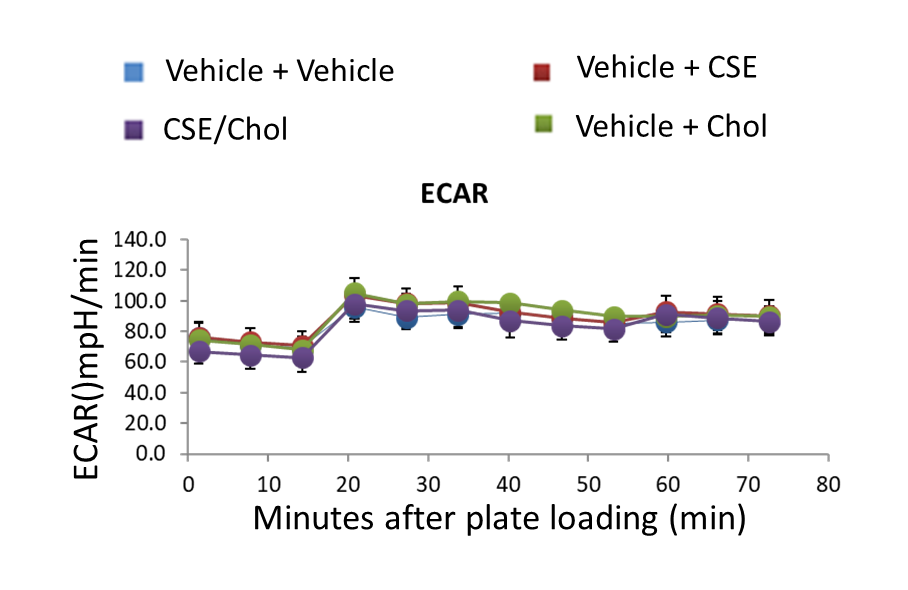
**4.** Extracellular acidification rate (ECAR) by Seahorse every 10 minutes for 80 minutes in human bronchial epithelial cells (HBEs) treated with vehicle, 6% cigarette smoke extract (CSE), 100μM cholesterol or combination (CSE/Chol).


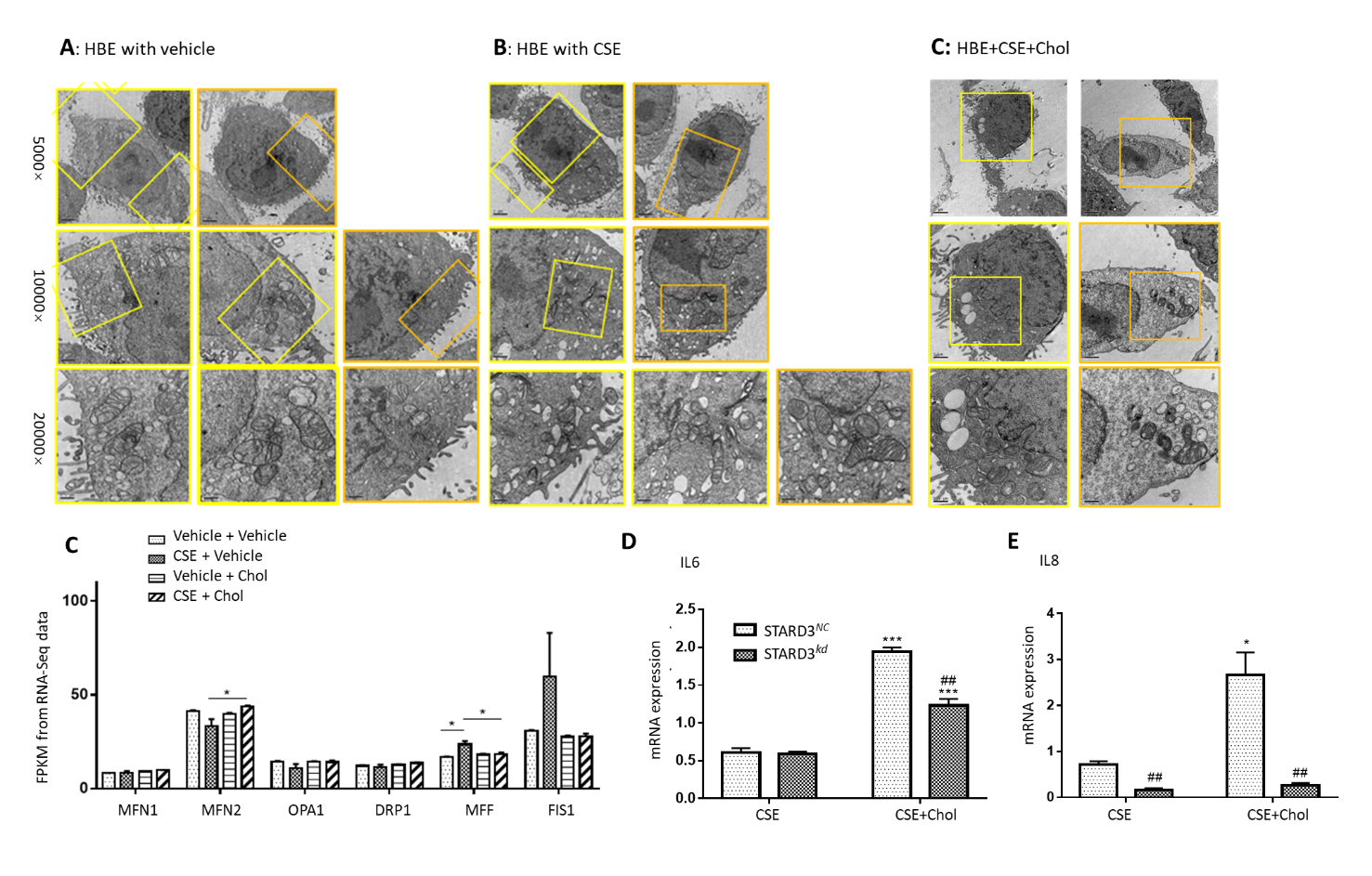


**Figure S5** Electron microscope of human bronchial epithelial cells (HBEs) stimulated with vehicle (A) and 6% cigarette smoke extract (CSE) (B) or combination (CSE/Chol) (C) for 24 hours. The transcriptional level of mitochondrial dynamics-related genes including mitofusin 1/2 (MFN1/2), optic atrophy 1 (OPA1), dynamin-related protein 1 (DRP1), mitochondrial fission factor (MFF), mitochondrial fission protein 1 (FIS1) in HBEs stimulated with vehicle, 6%CSE, 100μM cholesterol or CSE/Chol were analyzed from RNA-Seq data (D). The mRNA level of interleukin (IL) 6 (E), IL8 (F) were detected by RT-qPCR in STARD3 knockdown (STARD3*^kd^*) or negative control (STARD3*^NC^*) BEAS-2B cells stimulated with vehicle, 6%CSE, 100μM cholesterol or CSE/Chol. **p*<0.05; ***p*<0.01.* represents the intragroup comparison, # represents the intergroup comparison.

**
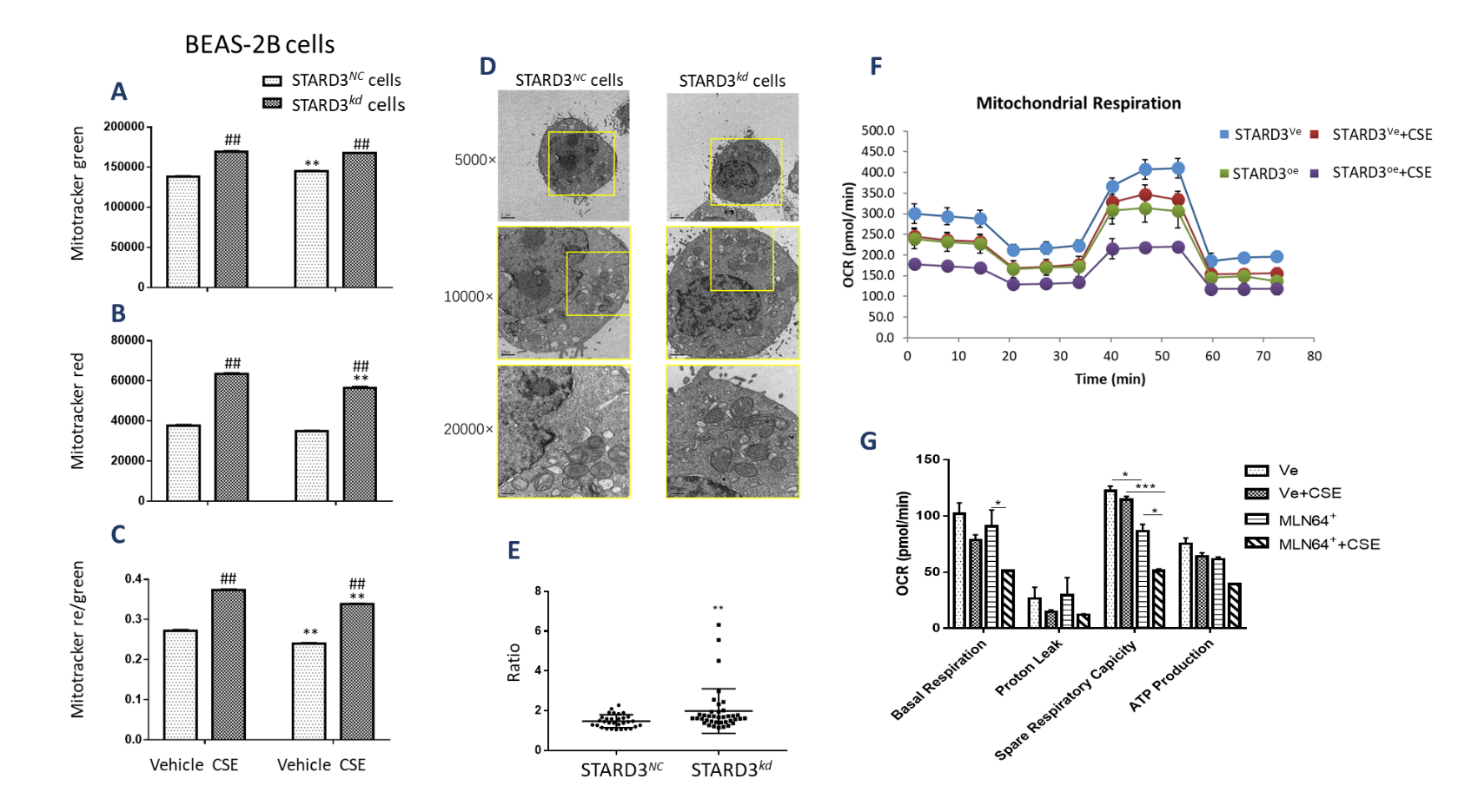
**

**Figure S6**. Mitochondrial mass by mitotracker green staining (A), mitochondrial membrane potential by mitotracker red staining (B) and standardized mitochondrial membrane potential by mitotracker red/green (C) were measured by flow cytometry in 6% cigarette smoke extract (CSE)-stimulated BEAS-2B cells with STARD3 knockdown (STARD3*^kd^*) or negative control (STARD*^NC^*). The morphology of mitochondria was observed by electron microscope (D) in STARD3*^kd^* and STARD*^NC^* treated with CSE/Chol and the ratio of length/width (D) in each mitochondrial was calculated by Image J. Oxygen consumption rate (OCR) (F) was measured for every 10 minutes for 80 minutes by Seahorse in STARD3*^oe^* or STARD3*^Ve^* treated with vehicle or 6% CSE, from which levels of basal respiration, proton leak, spare respiration capacity and ATP production were calculated (G). **p*<0.05; ***p*<0.01.* represents the intragroup comparison, # represents the intergroup comparison.


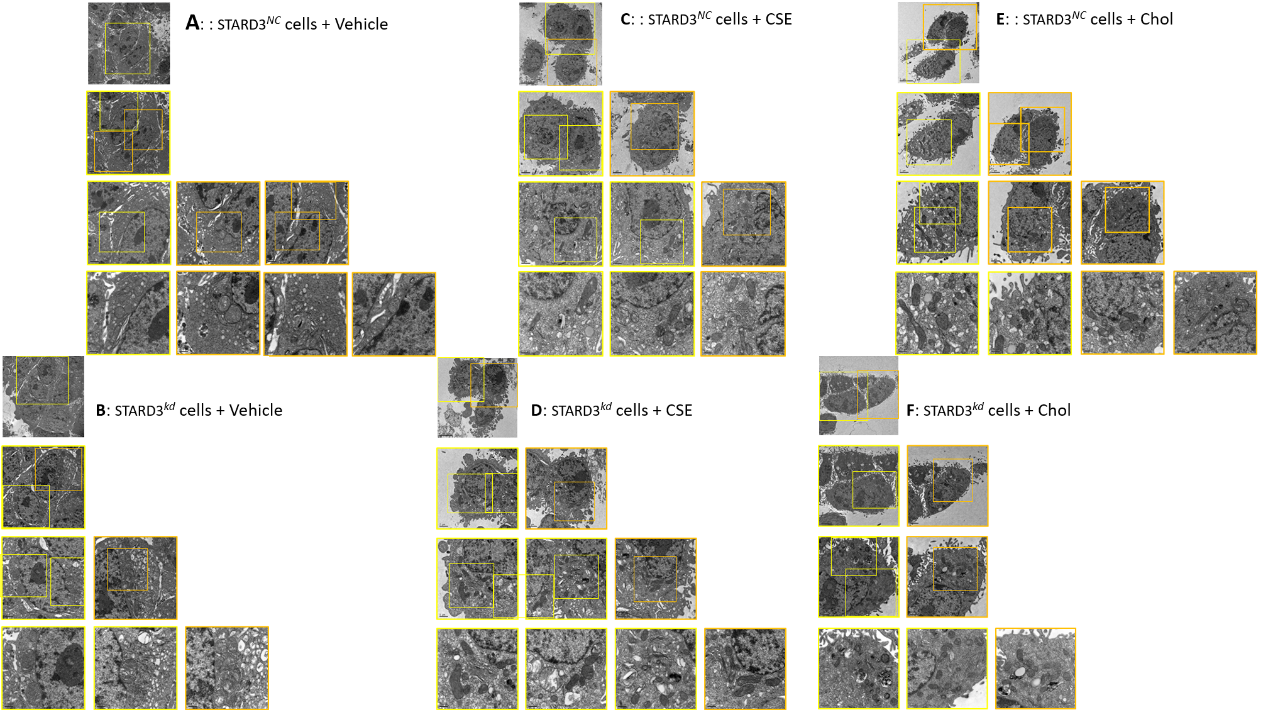


**Figure S7.** The ultrastructure of mitochondria in STARD3*^NC^*(A), STARD3*^kd^* (B), STARD3*^NC^* +6% cigarette smoke extract (CSE) (C), STARD3*^kd^* +6% CSE(D), STARD3*^NC^* +100μM cholesterol (E) and STARD3*^kd^* +100μM cholesterol (F) were also scanned by electron microscope. The magnification of each image was 3000X, 5000X, 10000X and 20000X from the top to the end.

**Table S1.** Demographic data of the clinical participants.

|  | Healthy control (N=21) | COPD(N=18) ^*^ |
| --- | --- | --- |
| Gender (Male, N) | 16 | 18 |
| Age (Years) | 38.14±11.02 | 66.89±10.14 |
| BMI | 24.10±4.00 | 23.38±4.05 |

^*^These patients were diagnosed as severe COPD ranked group D according to Global Initiative for Chronic Obstructive Lung Disease (GOLD).

**Table S2.** Lipid metabolism-targeted panel with 384 genes

**Table S3.** 242 differentially expressed genes (DEGs) of mitochondrial genes resulted from the RNA-seq data between cigarette smoke extract (CSE)-stimulated human bronchial epithelial cells (HBEs) and CSE/Chol co-stimulation HBE, which were filtered by Mitocarta 3.0.

| Gene Symbol | | | | | | | |
| --- | --- | --- | --- | --- | --- | --- | --- |
| NDUFS8 | MRPL13 | COX7C | ATP5PD | AURKAIP1 | NDUFB4 | DBI | MT-ND6 |
| TIMM9 | COX5B | SLC25A10 | ATP5MG | CHCHD7 | UQCR11 | LDHB | PIGBOS1 |
| SAMM50 | MRPL33 | MRPL22 | ATP5MC3 | MRPL14 | NDUFA1 | YBEY |  |
| NDUFV1 | NDUFB6 | MRPL47 | MRPL51 | COA3 | GUK1 | HINT1 |  |
| TIMM13 | SLC25A1 | CHCHD10 | TIMM17B | MRPS33 | NDUFAF2 | BID |  |
| MRPL40 | TOMM7 | SMDT1 | NIT2 | GADD45GIP1 | ECSIT | ARL2 |  |
| IDH3G | ECH1 | COX14 | TUFM | ROMO1 | PARL | DCXR |  |
| NDUFB5 | MRPL28 | NDUFA12 | NDUFAB1 | PRDX5 | DNAJC4 | VPS13D |  |
| SDHAF2 | TSFM | NDUFC2 | NDUFS3 | HIGD1A | ACSL6 | CYP11A1 |  |
| TXN2 | MRPL21 | SLIRP | NDUFB7 | ATP5MPL | DNAJC30 | SMIM8 |  |
| COX6C | NDUFB10 | MT-ATP6 | GLRX2 | CISD3 | DGUOK | FKBP8 |  |
| NDUFB9 | NDUFA2 | MT-CO2 | COX8A | BOLA1 | MT-ATP8 | ATP5MC2 |  |
| NDUFA6 | MRPL24 | NDUFA8 | SPR | GFER | MRPL52 | NTHL1 |  |
| COX17 | NDUFS5 | NDUFB11 | CHCHD1 | IMMP2L | MT-ND3 | SFXN4 |  |
| COX6B1 | MRPS7 | SPRYD4 | HSPE1 | MRPL39 | LYPLAL1 | PRDX2 |  |
| CLPP | MRPS12 | DECR1 | MRPL54 | MMAB | TMEM205 | ATP5MD |  |
| NDUFS4 | COX7A2 | MRPS26 | MRPL58 | LDHD | COA4 | C15orf48 |  |
| MRPL4 | TMEM126A | ADCK5 | TRIAP1 | C12orf65 | SDSL | PARK7 |  |
| MRPL55 | MRPL36 | ALKBH7 | MRM3 | BLOC1S1 | BAX | UQCC2 |  |
| MRPS14 | MARS2 | SSBP1 | NDUFC1 | NDUFAF8 | DTYMK | NME3 |  |
| NDUFA9 | FDXR | MRPL27 | NDUFA3 | ATP5MF | IDI1 | FASTK |  |
| COA6 | ALDH2 | HSCB | ECI1 | FAM162A | TSTD1 | COMTD1 |  |
| NDUFAF4 | ETHE1 | CISD1 | NDUFB3 | MACROD1 | PET100 | UQCC3 |  |
| TIMM8B | MRPL20 | MRPL41 | MRPL57 | TST | ISOC2 | HDHD3 |  |
| CMC2 | MRPS21 | MRPS6 | NDUFA4 | CHCHD5 | ATP5MC1 | GPX4 |  |
| SDHAF1 | ACAA2 | MRPL23 | NAXE | MT-ND2 | TSPO | DMAC1 |  |
| MRPL17 | NDUFB2 | SQOR | TMEM11 | CHCHD2 | MRPS36 | NDUFB1 |  |
| MRPL11 | MRPS34 | GCAT | COX7B | GPX1 | MCRIP2 | BBC3 |  |
| COX4I1 | ETFB | ECI2 | OXLD1 | FIS1 | SOD1 | BAD |  |
| NDUFS6 | ATP5IF1 | UQCRB | NDUFA11 | NUDT8 | BOLA3 | DMPK |  |

**Table S4.** Primers for RT-qPCR

|  | Forward | Reverse |
| --- | --- | --- |
| **human** |  |  |
| *ACTB* | AGCGAGCATCCCCCAAAGTT | GGGCACGAAGGCTCATCATT |
| *STARD3* | TACCAACACAGGCATCCGTAA | GCCAGGACAAAGATGTCGAAG |
| *IL6* | ACTCACCTCTTCAGAACGAATTG | CCATCTTGGAAGGTTCAGGTTG |
| *IL8* | ACTGAGAGTGATTGAGAGTGGAC | AACCCTCTGCACCCAGTTTTC |
| *IL1B* | ATGATGGCTTATTACAGTGGCAA | GTCGGAGATTCGTAGCTGGA |
| *HGMCR* | TGATTGACCTTTCCAGAGCAAG | CTAAAATTGCCATTCCACGAGC |
| *SREBP2* | CCTGGGAGACATCGACGAGAT | TGAATGACCGTTGCACTGAAG |
| *LDLR* | TCTGCAACATGGCTAGAGACT | TCCAAGCATTCGTTGGTCCC |
| *OPA1* | TGTGAGGTCTGCCAGTCTTTA | TGTCCTTAATTGGGGTCGTTG |
| *FIS1* | GATGACATCCGTAAAGGCATCG | AGAAGACGTAATCCCGCTGTT |
| *MFF* | CACCACCTCGTGTACTTACGC | GTCTGCCAACTGCTCGGATTT |
| *DRP1* | TTTGACACTTGTGGATTTGCCA | AGTGACAGCGAGGATAATGGA |
| *MFN2* | CTCTCGATGCAACTCTATCGTC | TCCTGTACGTGTCTTCAAGGAA |
| *MFN1* | TGGCTAAGAAGGCGATTACTGC | TCTCCGAGATAGCACCTCACC |
| **mouse** |  |  |
| *actb* | GGCTGTATTCCCCTCCATCG | CCAGTTGGTAACAATGCCATGT |
| *opa1* | TGGAAAATGGTTCGAGAGTCAG | CATTCCGTCTCTAGGTTAAAGCG |
| *drp1* | TTACGGTTCCCTAAACTTCACG | GTCACGGGCAACCTTTTACGA |
| *mfn2* | CTGGGGACCGGATCTTCTTC | CTGCCTCTCGAAATTCTGAAACT |
| *fis1* | TGTCCAAGAGCACGCAATTTG | CCTCGCACATACTTTAGAGCCTT |
| mff | AGCTGCCGCCACTTCTAATC | TGCATCTACCACAGTCATGTCA |
| mfn1 | ATGGCAGAAACGGTATCTCCA | CTCGGATGCTATTCGATCAAGTT |
